# Educational inequalities in Global Activity Limitation Indicator disability in 28 European Countries: Does the choice of survey matter?

**DOI:** 10.1007/s00038-018-1174-7

**Published:** 2018-11-26

**Authors:** Jose R. Rubio-Valverde, Wilma J. Nusselder, Johan P. Mackenbach

**Affiliations:** Department of Public Health, Erasmus Medical Center, P.O. Box 2040, 3000 CA Rotterdam, Netherlands

**Keywords:** GALI, Survey, Educational inequalities, International comparison

## Abstract

**Objectives:**

To assess the sensitivity of prevalence and inequality estimates of Global Activity Limitation Indicator (GALI) to the choice of survey in European countries.

**Methods:**

We use logistic regression to estimate adjusted risk ratios, quantifying differences in prevalence and educational inequalities, the impact of survey characteristics and Kendall’s tau to assess similarity in country rankings between surveys. We include the European Health Interview Survey (EHIS), European Social Survey (ESS) and European Union Statistics on Income and Living Conditions (EU-SILC).

**Results:**

EHIS estimates higher prevalence than EU-SILC 17% (men) and 23% (women), and ESS 24% (men) and 29% (women). Prevalence does not differ significantly between EU-SILC and ESS. EU-SILC estimates 52.5% (men) and 28.1% (women) higher inequalities than EHIS and 63.2% (men) and 32.7% (women) higher inequalities than ESS. Survey characteristics do not account for differences in prevalence or inequalities. Country rankings do not agree for prevalence or inequalities.

**Conclusions:**

Survey choice strongly impacts estimates of GALI prevalence and educational inequalities. Further study is necessary to understand these discrepancies. Caution is required when using these surveys for cross-country comparisons of (educational inequalities in) GALI disability.

**Electronic supplementary material:**

The online version of this article (10.1007/s00038-018-1174-7) contains supplementary material, which is available to authorized users.

## Introduction

Composite health metrics that combine data on mortality and health into a single measure of health expectancy are increasingly used to describe and understand changes in population health (Hyder et al. 2012; Brønnum-Hansen et al. 2015). The construction of these measures requires selecting from a range of health indicators. One of the most used indicators is the Global Activity Limitation Indicator (GALI), which can be combined with mortality data to estimate Healthy Life Years, the disability-free life expectancy measure that has been selected by the European Commission for standard use across Europe. GALI is part of the Minimum European Health module (MEHM). The importance of the GALI indicator is reflected in its presence in many national and cross-national surveys like the European Social Survey (ESS), the European Health Interview Survey (EHIS) and the European Statistics on Income and Living Conditions (EU-SILC).

The GALI has been shown to have good and sufficient concurrent and predictive validity, and reliability as well as fitting all conceptual characteristics of a global measure of participation restriction (Van Oyen et al. 2018). However, the indicator is self-reported, and is subject to variations in the tendency to report health problems (Berger et al. 2015b; Jürges 2007).

It is unknown whether different surveys that measure the GALI indicator lead to similar conclusions regarding prevalence and educational inequalities. Surveys differ in various characteristics, including sampling design, method of data collection, response rate, whether or not proxy respondents are allowed and the phrasing of the GALI question (EHLEIS 2011).

Prior research on self-reported health (SRH) based on three cross-national surveys in 10 European countries (EU-SILC; Survey of Health, Aging and Retirement in Europe (SHARE) and ESS) showed that the prevalence of less-than-good SRH varies significantly across surveys and that differences between surveys in response rate, sample size and collection mode contributed to these differences (Croezen et al. 2016). A second study by Toch-Marquardt (2017) on occupational inequalities in SRH comparing four European surveys (ESS; EU-SILC; European Working Conditions Survey (EWCS); and International Social Survey Programme (ISSP), found that both prevalence and occupational inequalities in SRH vary significantly by survey, and was unable to detect regional patterns in inequalities that are consistent across surveys. For smoking, prevalence and educational inequalities also vary by survey (Kulik et al. 2014).

For the GALI indicator, evidence is lacking whether the prevalence levels and educational differences differ between surveys and whether survey characteristics could explain possible differences and hence inform the choice which survey to use, or to allow obtaining a pooled estimate for a specific combination of survey characteristics. Therefore, the primary aim of this paper is to assess whether three widely used nationally representative European surveys provide similar or different estimates of prevalence of GALI disability and of educational inequalities in GALI disability in Europe. The secondary aim is to assess the role of survey characteristics in these variations between the surveys.

## Methods

### Data

#### Description of surveys

The European Health Interview Survey (EHIS) mainly gathers health-related indicators. It has four modules including variables of health status, healthcare use, health determinants and socioeconomic background. The survey targets individuals above 15 years old living in private households. It was implemented from 2006 to 2009 in 17 EU member states and is repeated every 5 years. We included all 15 countries from the first wave of EHIS, with a total sample size of 125,293 persons.

The European Social Survey (ESS) is a biennial cross-national survey starting from 2001. It surveys beliefs, attitudes and behavior patterns of populations of more than 30 countries. The samples are representative of all individuals over 15 years old living in private households and have a minimum size of 1.500 individuals, except for countries with less than 2 million inhabitants. We included data for 27 countries for 2008, 2010 and 2012, for a total of 103,829 individuals (ESS 2016).

The European Union Statistics on Living Conditions (EU-SILC) survey provides annual data on variables on poverty, income, social exclusion and living conditions. The survey was launched in 2003, and has extended its coverage to the 28 member states of the enlarged European Union. The target population is all private households and their members living in the country’s territory. All household members are surveyed, and only those above 16 years are interviewed. EU-SILC provides both cross-sectional and longitudinal data. We have pooled the cross-sectional data for the years 2008 and 2012. Considering the rotating panel structure (Eurostat 2016), intermediate years are excluded to avoid including subject more than once. We included 28 countries from EU-SILC, with a total sample size of 603,785.

Countries included in our analysis must be present in at least two of the three surveys. We restricted the analysis to persons between 30 and 79 years old because below age 30 not everybody has completed his/her education, and above age 80 an increasing fraction of the population is institutionalized. ESS, EU-SILC and EHIS include only persons living in private households. Because of lack of sample representativeness in EU-SILC, we excluded Luxembourg and Malta (Cambois et al. 2016b).

The countries included in our analysis were Denmark, Finland, Norway, Sweden, the UK, Ireland, the Netherlands, Belgium, Germany, Austria, Switzerland, France, Spain, Italy, Greece, Cyprus, Slovenia, Croatia, Czech Republic, Slovakia, Hungary, Poland, Bulgaria, Romania, Latvia, Lithuania and Estonia. For clarity of presentation, we present the countries according to geographical region.

#### Measure of disability

The GALI question is: “For at least the past 6 months, to what extent have you been limited in activities people usually do?”. EHIS used the standard version of the question across all countries, and ESS omitted the time reference in the question. Countries in EU-SILC had more diverse implementation of the question, with 13 using the standard GALI question in 2008, and variations including the omission of the time frame, changing the generic “activities people do” for a more personal reference; and breaking the single question into parts. The response categories were similar across surveys, with three potential responses (“Yes, a lot”; “Yes, some”; “No”). For our analysis, we combined yes categories.

#### Measure of socioeconomic status

All three surveys provided ISCED-97 educational attainment. We combined the ISCED categories to form 3 levels of education: low, medium and high, corresponding to ISCED categories 0–2, 3–4 and 5–6, respectively.

#### Survey characteristics

We collected information on survey characteristics from technical and quality reports of the different surveys: individual response rate (%), sample size (in thousands), sampling design in three categories (simple random one/multistage; stratified random one/multistage; stratified systematic one/multistage), proxy respondents (as a binary variable) and collection mode in three categories (present interviewer (PAPI—Paper and Pencil Interviewing—and CAPI—Computer-Assisted Personal Interviewing), remote interviewer (CATI—Computer-Assisted Telephone Interview) and other (including countries that use several modes of data collection and Germany in EU-SILC, which uses a self-administered questionnaire)). Information on survey characteristics is presented in Online Resource 1.

### Statistical analyses

#### Prevalence

We calculated for each country and survey age-standardized prevalence of GALI disability by gender, using the 2013 European Standard Population.

We used logistic regression and the post-estimation command *adjrr* in STATA, and obtained adjusted risk ratios (ARRs) for pairs of surveys (Norton et al. 2013). These regression models included age category (30–34; 15–39;…75–79) and survey as independent variables and were stratified by country and gender. The ARRs indicate whether differences in prevalence exist between surveys relative to the baseline survey.

Next, we pooled data across countries. This second set of regression models additionally included country (with 27 levels) and education. The ARR indicates whether on average differences in prevalence exist between surveys while controlling for country and education. Standard errors were clustered at the country level to account for potential correlation of individuals within a country. We repeated this analysis, stratified by education to assess if survey variation in prevalence of GALI disability is different across educational groups.

#### Educational inequalities

Similar to the prevalence analyses, we started with separate analyses for country, gender and survey. We used logistic regression models with age category and education as independent variables. We derived ARRs for low versus high educated to compare the variation in educational inequalities in GALI disability for individual countries for each survey and gender. To test whether the educational inequalities are significantly different across pairs of surveys within a country, we pooled data for each pair of survey, added a survey interaction with education and conducted likelihood ratio (LR) tests to compare between models with and without this interaction term.

Next, we pooled data across countries to examine the average difference of the educational inequalities across the three surveys. The *adjrr* command calculated educational ARRs using the coefficients for education, survey and the interaction between them and indicates survey-specific educational ARR, when controlling for age category and country.

#### Survey characteristics

We extended the survey–country pooled models for prevalence and inequalities with survey characteristics to assess to what extent variations in survey characteristics explain differences between surveys. This involved assessing the significance of each survey characteristic individually using Wald tests. We then included all statistically significant survey characteristics and the interactions between these survey characteristics and education in the final model.

We assessed whether the inclusion of the survey characteristics and their interactions altered the derived ARR for differences in prevalence between surveys. These regression models combined survey characteristics at the survey level with individual level data, but the *adjrr* STATA command to derive ARRs has not been adapted for the multi-level setting. We conducted robustness analyses using multi-level logistic regression, with country at the higher level and survey nested within country (included as a random effect), and compared the results with the standard logistic regression. Taking into account the multi-level structure of the data did not alter our results (Online Resource 4).

#### Ranking comparison

We used age-standardized prevalences and country educational inequalities (ARRs) to create rankings of countries in terms of the two outcomes. We paired surveys and restricted the rank comparison only to countries present in both surveys. For each pair of rankings, we estimated Kendall’s tau and its associated *p* value. A value of − 1 implies perfect reversal of the rankings, while a value of 1 implies perfect agreement. We chose Kendal’s tau over other rank correlation measures like Spearman’s correlation because it has been shown to be slightly more robust and efficient (Croux and Dehon 2010). The ranking comparisons were stratified by gender.

We focused on relative educational inequalities in GALI prevalence. All analyses were repeated for absolute educational inequalities (Online Resource 2).

## Results

### Prevalence of GALI disability

Table 1 shows the age-standardized GALI prevalence and the ARRs by survey for each country, stratified by gender. Confidence intervals can be found in Online Resource 3.

**Table 1:** Age-standardized disability prevalence (age 30–79) in 28 European countries by gender and survey (European Health Interview Survey 2006–2009; European Social Survey 2008, 2010, 2012; European Union Statistics on Income and Living conditions 2008, 2012) and adjusted risk ratios comparing prevalence estimates between surveys

	Prevalence (%)^a^			Adjusted Risk Ratios (ARRs)^b^				Prevalence^a^			Adjusted Risk Ratios (ARRs)^b^		
	EU-SILC	EHIS	ESS	EHIS versus EU-SILC	ESS versus EU-SILC	ESS versus EHIS		EU-SILC	EHIS	ESS	EHIS versus EU-SILC	ESS versus EU-SILC	ESS versus EHIS
*Males*							*Females*						
Finland	29	–	33	**–**	**1.10**	–	Finland	32	–	32	**–**	0.96	–
Sweden	13	–	25	**–**	**1.8**	–	Sweden	19	–	28	**–**	**1.49**	–
Norway	11	–	24	**–**	**2.02**	–	Norway	18	–	29	**–**	**1.58**	–
Denmark	24	–	23	**–**	0.97	–	Denmark	29	–	29	**–**	1.02	–
UK	19	–	24	**–**	**1.21**	–	UK	22	–	26	**–**	**1.15**	–
Ireland	19	–	18	**–**	0.92	–	Ireland	20	–	15	**–**	**0.77**	–
Netherlands	23	–	21	–	**0.9**	–	Netherlands	31	–	30	–	0.95	–
Belgium	20	19	23	**0.92**	**1.11**	**1.21**	Belgium	23	24	26	**0.93**	**1.09**	**1.07**
Germany	33	–	30	–	**0.91**	–	Germany	33	–	30	–	**0.87**	–
Austria	28	34	–	**1.19**	**–**	**–**	Austria	28	35	–	**1.22**	–	–
Switzerland	21	–	17	**–**	**0.95**	–	Switzerland	24	–	20	**–**	**0.85**	–
France	22	24	23	**1.09**	1.03	0.95	France	23	27	24	**1.11**	0.99	**0.89**
Spain	20	21	12	1.04	**0.62**	**0.59**	Spain	23	28	18	**1.18**	**0.81**	**0.68**
Portugal	23	–	14	**–**	**0.6**	–	Portugal	29	–	17	–	**0.62**	–
Italy	24	–	16	**–**	**0.71**	–	Italy	27	–	18	–	**0.71**	–
Greece	17	17	10	0.97	**0.6**	**0.61**	Greece	20	26	16	**1.21**	**0.82**	**0.67**
Cyprus	21	18	16	**0.89**	**0.78**	0.88	Cyprus	23	22	22	1	0.99	0.98
Slovenia	29	36	30	**1.21**	1.03	**0.85**	Slovenia	31	41	30	**1.28**	0.95	**0.74**
Croatia	21	–	28	**–**	**1.25**	–	Croatia	21	–	24	**–**	**1.12**	–
Czech Rep.	21	30	26	**1.37**	**1.23**	0.9	Czech Rep.	22	30	32	**1.29**	**1.4**	1.09
Slovakia	35	42	24	**1.21**	**0.7**	**0.57**	Slovakia	39	47	27	**1.19**	**0.72**	**0.62**
Hungary	26	40	29	**1.47**	**1.08**	**0.74**	Hungary	28	45	30	**1.46**	1.03	**0.7**
Poland	23	27	28	**1.13**	**1.2**	1.06	Poland	23	28	31	**1.17**	**1.26**	**1.08**
Bulgaria	15	20	14	**1.36**	0.91	**0.67**	Bulgaria	16	25	16	**1.53**	1.01	**0.66**
Romania	21	22	17	1.05	**0.77**	**0.74**	Romania	26	29	21	1.06	**0.76**	**0.71**
Latvia	30	47	36	**1.51**	**1.19**	**0.79**	Latvia	31	51	41	**1.46**	**1.26**	**0.87**
Lithuania	24	–	31	**–**	**1.29**	–	Lithuania	25	–	39	**–**	**1.41**	–
Estonia	32	38	26	**1.14**	**0.8**	**0.7**	Estonia	31	41	24	**1.2**	**0.76**	**0.64**
Total	23	27	23	**1.29**	0.97	**0.75**		26	31	26	**1.30**	0.97	**0.75**

^a^Prevalence rates were standardized using the 2013 European Standard population

^b^The risk ratios are derived after fitting logistic regressions using the post-estimation command a*djrr* in STATA. The models are stratified by country and include age and survey as covariates. The ARRs are derived from the survey coefficients. All models include robust standard errors. Prevalences with 95% CIs are included in Table A2 in ESM. Significant values in bold (*p* < 0.05)

There is substantial variation in prevalence between surveys. For men, the ARRs using as reference EU-SILC indicate that EHIS provides statistically significantly higher prevalence estimates for 11 of the 15 countries, while ESS yields lower prevalence for 2 countries (Belgium, Cyprus) and no significant difference for the 2 remaining countries (Greece, Romania). For women, EHIS estimates higher prevalence than EU-SILC in 12 countries, with the 3 remaining countries showing no statistically significant differences between the two surveys. When comparing ESS with EU-SILC, the results are diverse. For men in 11 of the 27 countries, ESS yields significantly higher prevalence estimates; in 10 lower and in 6 not statistically different. Women display the same pattern.

When comparing ESS and EHIS, for men, ESS produces higher prevalence estimates for one country (Belgium), lower prevalence for 9 countries and no statistically significant difference for 4 countries. Women display a similar pattern, with EHIS estimating higher prevalence also for Poland.

Table 2 (Model 1) presents the average across all countries of the patterns described above for men and women. For men, as compared to EU-SILC, EHIS estimates on average 17% higher prevalence (ARR = 1.17, CI 1.09, 1.25), and ESS a non-statistically significant 6% lower prevalence (ARR = 0.94, CI 0.84, 1.05). For women, EHIS estimates 23% higher prevalence (ARR = 1.23, CI 1.06, 1.30) and a non-significant 5% lower prevalence (ARR = 0.95, CI 0.85, 1.06). The average difference in prevalence for ESS is not statistically different from EHIS.

**Table 2:** Adjusted risk ratios of Global Activity Limitation Indicator disability for survey (ref = 1, European Union Statistics on Income and Living Conditions) and survey characteristics—based on country/survey pooled data (ages 30–79) for 28 European countries (European Health Interview Survey 2006–2009; European Social Survey 2008, 2010, 2012; European Union Statistics on Income and Living conditions 2008, 2012)

	Model 1	Model 2	Model 3	Model 4	Model 5	Model 6
	Risk ratio [95% CI]	Risk ratio [95% CI]	Risk ratio [95% CI]	Risk ratio [95% CI]	Risk ratio [95% CI]	Risk ratio [95% CI]
Men						
Survey						
EU-Survey Income Living Conditions (EU-SILC)	1.00 [ref]	1.00 [ref]	1.00 [ref]	1.00 [ref]	1.00 [ref]	1.00 [ref]
European Health Interview Survey (EHIS)	**1.17** [1.09, 1.25]	**1.22** [1.10, 1.33]	**1.14 [**1.08, 1.21]	**1.15** [1.07, 1.24]	**1.18** [1.10, 1.26]	**1.17** [1.09, 1.25]
European Social Survey (ESS)	0.94 [0.84, 1.05]	0.93 [0.81, 1.05]	0.85 [0.71, 1.01]	0.90 [0.81, 1.01]	0.85 [0.70, 1.01]	0.95 [0.78, 1.16]
Education						
Low	**1.77** [1.68, 1.85]	**1.76** [1.67, 1.83]	**1.76** [1.68, 1.85]	**1.76** [1.68, 1.85]	**1.76** [1.68, 1.85]	**1.77** [1.68, 1.85]
Medium	**1.35** [1.27,1.43]	**1.34** [1.25 1.43]	**1.35** [1.27, 1.43]	**1.34** [1.27, 1.42]	**1.34** [1.27, 1.42]	**1.35** [1.27, 1.43]
High	1.00 [ref]	1.00 [ref]	1.00 [ref]	1.00 [ref]	1.00 [ref]	1.00 [ref]
Survey characteristics						
Response rate		0.99 [0.93, 1.05]				
Sample size (× 1000)			1.00 [0.98, 1.02]			
Sampling design						
Simple, random				1.00 [ref]		
Stratified, random				0.82 [0.65, 1.04		
Stratified, systematic				0.81 [0.64, 1.04]		
Collection mode^a^						
CAPI and PAPI					1.00 [ref]	
CATI					0.82 [0.65, 1.04]	
Other					0.81 [0.64, 1.04]	
Proxy allowed						
No						1.00 [ref]
Yes						1.02 [0.82, 1.26]
*N*	387,228	295,064	387,011	387,011	387,011	387,011
Wald test	–	0.76	0.08	0.29	0.02	0.83
Women						
Survey						
EU-Survey Income Living Conditions (EU-SILC)	1.00 [ref]	1.00 [ref]	1.00 [ref]	1.00 [ref]	1.00 [ref]	1.00 [ref]
European Health Interview Survey (EHIS)	**1.23** [1.06, 1.30]	**1.23** [1.15, 1.34]	**1.21** [1.14, 1.28]	**1.21** [1.13, 1.29]	**1.18** [1.10, 1.26]	**1.22** [1.16, 1.30]
European Social Survey (ESS)	0.95 [0.85, 1.06]	0.92 [0,82, 1.03]	0.93 [0.81, 1.10]	0.88 [0.77, 1.01]	0.85 [0.70, 1.01]	0.89 [0.76, 1.04]
Education						
Low	**1.65** [1.58, 1.72]	**1.63** [1.55, 1.70]	**1.65** [1.57, 1.73]	**1.65** [1.57, 1.73]	**1.65** [1.57, 1.72]	**1.65** [1.57, 1.72]
Medium	**1.25** [1.18, 1.27]	**1.25** [1.21, 1.30]	**1.25** [1.21, 1.30]	**1.25** [1.20, 1.29]	**1.25** [1.21, 1.29]	**1.25** [1.21., 1.30]
High	1.00 [ref]	1.00 [ref]	1.00 [ref]	1.00 [ref]	1.00 [ref]	1.00 [ref]
Survey characteristics						
Response rate		1.00 [0.99, 1.00]				
Sample size (× 1000)			0.99 [0.98, 1.01]			
Sampling design						
Simple, random				1.00 [ref]		
Stratified, random				0.91 [0.76, 1.11]		
Stratified, systematic				0.90 [0.73, 1.11]		
Collection mode^a^						
CAPI and PAPI					1.00 [ref]	
CATI					**0.83** [0.70, 0.96]	
Other					0.87 [0.72, 1.03]	
Proxy allowed						
No						1.00 [ref]
Yes						0.88 [0.72, 1.06]
*N*	445,438	337,395	445,438	445,438	445,438	445,438
Wald test	–	0.82	0.70	0.19	0.03	0.34

Significance in bold (*p* < 0.05)

Model 1 includes all pooled data for countries and surveys, stratified only by sex. The model is ${\text{logit}}({\text{GALI}}_{\text{iksce}} ) = \beta_{0} + \beta_{k} \;{\text{Age}} + \beta_{s} \;{\text{Survey}}_{s} + {\text{Country}}_{c} + \beta_{e} {\text{Education}}_{e}$

Models 2–6 use ${\text{logit}}\left( {{\text{GALI}}_{\text{ikesc}} } \right) = \beta_{k} {\text{Age}} + \beta_{s} {\text{Survey}}_{s} + \beta_{e} {\text{Education}}_{e} + \beta_{c} {\text{Country}}_{c} + \beta_{n} {\text{SurveyChar}}_{n}$. The Wald tests compare baseline Model 1 with Model 2–6

EHIS—European Health Interview Survey (2006/2009); ESS—European Social Survey (2008, 2010, 2012); EU-SILC—EU Statistics on Income and Living Conditions (2008, 2012)

^a^CAPI, Computer-Assisted Personal Interview; PAPI, Pencil and Paper Interview; CATI, Computer-Assisted Telephone Interview

The stratified analysis by education shows that ESS estimates statistically significantly lower prevalence than EU-SILC and EHIS for the low educated group (Table 3). The difference as compared to EU-SILC is 12% (ARR = 0.88, 95% CI 0.81, 0.97) for men and 9% (ARR = 0.91, 95% CI 0.83, 0.99) for women. The results for other educational levels are consistent with the results from the model with all educational levels showing higher prevalence for EHIS than EU-SILC, although the difference is larger for the high educated (ARR = 1.31, 95% CI 1.19, 1.42) than for the low educated (ARR = 1.07, 95% CI 1.01, 1.13). Women display a similar pattern.

**Table 3:** Prevalence analysis stratified by education

Survey	Model A Low educated		Model B Medium educated		Model C High educated	
Men						
EU-Survey Income Living Conditions (EU-SILC)	1.00	Ref	1.00	Ref	1.00	Ref
European Health Interview Survey (EHIS)	**1.07**	[1.01, 1.13]	**1.22**	[1.13, 1.32]	**1.31**	[1.19, 1.42]
European Social Survey (ESS)	**0.88**	[0.81, 0.97]	0.97	[0.83, 1.11]	1.07	[0.95, 1.22]
* n*	119,290		180,158		87,563	
Women						
EU-Survey Income Living Conditions (EU-SILC)	1.00	Ref	1.00	Ref	1.00	Ref
European Health Interview Survey (EHIS)	**1.15**	[1.10, 1.21]	**1.35**	[1.16, 1.56]	**1.36**	[1.19, 1.54]
European Social Survey (ESS)	**0.91**	[0.83, 0.99]	0.97	[0.84, 1.12]	1.06	[0.81, 1.26]
* n*	156,327		190,606		98,505	

Adjusted risk ratio of disability prevalence (between surveys) for the pooled dataset, stratified by education for men and women (ages 30–79) for 28 European countries (European Health Interview Survey 2006–2009; European Social Survey 2008, 2010, 2012; European Union Statistics on Income and Living conditions 2008, 2012)

Significance at the 5% level in bold

Models are stratified by sex and education

The models presented correspond to ${\text{logit}}\left( {{\text{Global}}\;{\text{Activity}}\;{\text{Limitation}}\;{\text{Indicator}} - {\text{GALI}}} \right) = \beta_{k} {\text{Age}}_{k} + \beta_{s} {\text{Survey}}_{s} + \beta_{c} {\text{Country}}_{c}$

EHIS—European Health Interview Survey (2006/2009); ESS—European Social Survey (2008, 2010, 2012); EU-SILC—EU Statistics on Income and Living Conditions (2008, 2012) for 28 European countries (Finland, Sweden, Norway, Denmark, the UK, Ireland, the Netherlands, Belgium, Germany, Austria, Switzerland, France, Spain, Portugal, Italy, Greece, Cyprus, Slovenia, Croatia Czech Republic, Slovakia, Hungary, Poland, Bulgaria, Romania, Latvia, Lithuania, Estonia)

### Educational inequalities between surveys

**Fig. 1:**
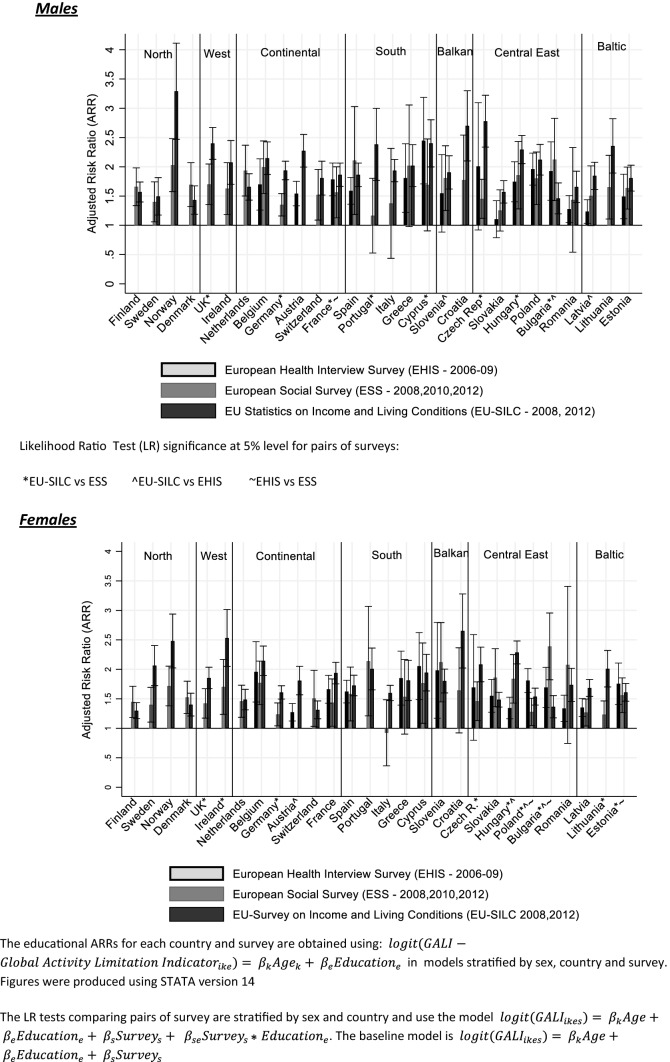
Relative educational inequalities (low vs. high educated aged 30–79) in disability prevalence in 28 European countries by gender and survey (European Health Interview Survey 2006–2009; European Social Survey 2008, 2010, 2012; European Union Statistics on Income and Living conditions 2008, 2012)

Figure 1 shows educational differences in GALI prevalence by country, survey and gender (CIs are presented in Online Resource 5). For both genders and most countries, the ARRs are substantially higher than 1, indicating a higher prevalence of GALI disability among the low educated as compared to the high educated, although several exceptions exist. These include Czech Republic (EHIS men and women), Slovenia (EHIS men), Slovakia (EHIS and ESS men), Italy (ESS men; EHIS women), Portugal (ESS men), Cyprus (ESS men), Greece (ESS men; EHIS women), Romania (ESS men; EHIS women) and Croatia (EHIS women).

The average ARR for men for all countries combined for EU-SILC is 1.93 (95% CI 1.79–2.07) as compared to 1.61 (95% CI 1.46–1.66) for EHIS (Table 4, Model 1). This corresponds to 52.5% higher educational differences in EU-SILC than in EHIS. When comparing EU-SILC and ESS, estimates of ARR are 63.2% higher than in ESS (1.93/1.57)/(1.57–1.00). For women in EU-SILC, the differences were 28.1% higher for EU-SILC (1.73–1.57)/(1.57–1.00) as compared to EHIS and 32.7% higher as compared to ESS (1.73–1.55)/(1.55–1.00).

**Table 4:** Educational Inequalities (low vs. high) in disability prevalence (risk ratio and 95% confidence intervals) by survey—based on country/survey pooled data (ages 30–79) for 28 European countries (European Health Interview Survey 2006–2009; European Social Survey 2008, 2010, 2012; European Union Statistics on Income and Living conditions 2008, 2012)

	Model 1	Model 2	Model 3	Model 4	Model 5	Model 6
	Risk ratio [95% CI]	Risk ratio [95% CI]	Risk ratio [95% CI]	Risk ratio [95% CI]	Risk ratio [95% CI]	Risk ratio [95% CI]
Men						
Survey-Specific Ed. Inequalities						
European Health Interview Survey (EHIS)	1.61 [1.46, 1.78]	1.66 [1.44, 1.91]	1.60 [1.51, 1.67]	1.57 [1.42, 1.73]	1.58 [1.42, 1.75]	1.55 [1.38, 1.73]
European Social Survey (ESS)	1.57 [1.48, 1.66]	1.56 [1.48, 1.65]	1.58 [1.44, 1.69]	1.56 [1.43, 1.70]	1.57 [1.44, 1.68	1.64 [1.52, 1.78]
EU-Statistics Income Living Conditions (EU-SILC)	1.93 [1.79, 2.07]	1.94 [1.81, 2.08]	1.91 [1.77, 2.05]	1.94 [1.81, 2.08]	1.93 [1.82, 2.04]	1.89 [1.74, 2.04]
Survey characteristics						
Response rate		1.00 [0.99, 1.00]				
Sample size (× 1000)			1.00 [0.98, 1.02]			
Sampling design						
Simple, random				1.00 [ref]		
Stratified, random				082 [0.64, 1.04		
Stratified, systematic				0.80 [0.63, 1.03]		
Collection mode^a^						
CAPI and PAPI					1.00 [ref]	
CATI					**0.83** [0.69, 0.98]	
Other					0.89 [0.78, 1.02]	
Proxy allowed						
No						1.00 [ref]
Yes						1.02 [0.83, 1.26]
*N*	387,228	295,064	387,011	387,011	387,011	387,011
Wald test	–	0.78	0.75	0.11	0.02	0.37
Women						
Survey-specific Ed. inequalities						
European Health Interview Survey (EHIS)	1.57 [1.41, 1.75]	1.60 [1.41, 1.75]	1.57 [1.44, 1.72]	1.49 [1.43, 1.72]	1.51 [1.43, 1.73]	1.51 [1.34, 1.71]
European Social Survey (ESS)	1.55 [1.41, 1.69]	1.54 [1.41, 1.69]	1.55 [1.42, 1.70]	1.57 [1.42, 1.73]	1.52 [1.28, 1.51]	1.60 [1.45, 1.77]
EU-Statistics Income Living Conditions (EU-SILC)	1.73 [1.61, 1.87]	1.72 [1.61, 1.87]	1.72 [1.59, 1.86]	1.74 [1.61, 1.75]	1.77 [1.61, 1.85]	1.70 [1.55, 1.87]
Survey characteristics						
Response rate		1.00 [0.99, 1.00]				
Sample size (× 1000)			1.00 [0.99, 1.01]			
Sampling design						
Simple, random				1.00 [ref]		
Stratified, random				0.92 [0.75, 1.08]		
Stratified, systematic				0.90 [0.66, 1.12]		
Collection mode^a^						
CAPI and PAPI					1.00 [ref]	
CATI					0.89 [0.75, 1.05]	
Other					0.88 [0.78, 1.00]	
Proxy allowed						
No						1.00 [ref]
Yes						0.88 [0.72, 1.06]
*N*	445,438	337,695	445,438	442,953	445,438	445,438
Wald test	–	0.84	0,72	0.22	0.02	0.15

Model 1 includes all pooled data for countries and surveys, stratified only by sex. The model is ${\text{logit}}({\text{GALI}}_{\text{iksce}} ) = \beta_{0} + \beta_{k} {\text{Age}} + \beta_{s} {\text{Survey}}_{s} + {\text{Country}}_{c} + \beta_{e} {\text{Education}}_{e} \; \beta_{se} {\text{Survey}}_{s} *{\text{Education}}_{e}$

Models 2–6 use ${\text{logit}}\left( {{\text{GALI}}_{\text{ikesc}} } \right) = \beta_{k} {\text{Age}} + \beta_{s} {\text{Survey}}_{s} + \beta_{e} {\text{Education}}_{e} + \beta_{c} {\text{Country}}_{c} + \beta_{se} {\text{Survey}}_{s} *{\text{Education}}_{e} + \beta_{n} {\text{SurveyChar}}_{n} (6) + \beta_{ne} {\text{SurveyChar}}_{n} *{\text{Education}}_{e}$. The Wald tests compare baseline Model 1 with Model 2–6

EHIS—European Health Interview Survey (2006/2009); ESS—European Social Survey (2008,2010,2012); EU-SILC—EU Statistics on Income and Living Conditions (2008, 2012). For 28 European countries (Finland, Sweden, Norway, Denmark, the UK, Ireland, the Netherlands, Belgium, Germany, Austria, Switzerland, France, Spain, Portugal, Italy, Greece, Cyprus, Slovenia, Croatia Czech Republic, Slovakia, Hungary, Poland, Bulgaria, Romania, Latvia, Lithuania, Estonia)

^a^CAPI (Computer-Assisted Personal Interview); PAPI(Pencil and Paper Interview); CATI(Computer-Assisted Telephone Interview)

### Survey characteristics

We find a statistically significant association between collection mode and GALI prevalence only; none of the other survey characteristics is associated with GALI prevalence. Relative to present interviewer (PAPI and CAPI), remote interviewer (CATI) is associated with a lower GALI prevalence for men (ARR = 0.81, 95% CI 0.68–0.99) and women (ARR = 0.83, 95% CI 0.70–0.96). Controlling for collection mode does not change the difference between EHIS and EU-SILC as can be seen by comparing the ARR for survey according to Model 1 (ARR = 1.17, 95% CI 1.09, 1.25) with that of Model 5 (ARR = 1.18, 95% CI 1.10, 1.26) for men and by comparing Model 1 (ARR = 1.23, 95% CI 1.06, 1.30) with Model 5 (ARR = 1.25, 95% CI 1.16, 1.31) for women.

For the educational inequalities, only the inclusion of collection mode has a modest impact on the survey-specific inequalities (Table 4). Comparing the ARRs between the models with and without adjustment for this survey characteristic shows no reduction for EU-SILC and ESS and a small reduction for EHIS (ARR: 158 vs. 1.61) for men. For women, adjusting for survey characteristics shows small reduction for EHIS (ARR 1.51 vs. 1.57) and ESS (ARR 1.52 vs. AR 1.55), but a small increase for EU-SILC (1.77 vs. 1.73).

### Rank comparison

Table 5 shows that the ranks of both prevalence and inequalities given by the surveys do not agree, with correlations close to 0 in most cases. For the prevalence, only the rank comparison between EHIS and ESS for men is close to being statistically significant (Tau = 0.36 and *p* value = 0.07). For the compassion of educational inequalities, the exception is the rank comparison between ESS and EHIS which is statistically significantly correlated at the 5% level, though with relatively low Kendall’s tau of 0.40.

**Table 5:** Kendal’s Tau Correlations and associated *p* value comparing country rank agreement (risk ratio) between surveys (European Health Interview Survey 2006–2009; European Social Survey 2008, 2010, 2012; European Union Statistics on Income and Living conditions 2008, 2012) for prevalence and educational inequalities—based on country/survey pooled data (ages 30–79) for 28 European countries

Prevalence (Kendall’s tau^b^ and *p* value)				Educational inequalities (Kendall’s Tau^b^ and *p* value)			
Pair of Surveys	Kendall’s Tau (− 1 to 1)	*p* value	*n* ^a^	Pair of surveys	Kendall’s Tau (− 1 to 1)	*p* value	*n* ^a^
*Men*				*Men*			
EU-SILC versus ESS	0.13	0.31	27	EU-SILC versus ESS	− 0.06	0.67	27
EU-SILC versus EHIS	− 0.16	0.42	15	EU-SILC versus EHIS	0.12	0.55	15
EHIS versus ESS	0.36	0.07	14	EHIS versus ESS	0.40	**0.04**	14
*Women*				*Women*			
EU-SILC versus ESS	− 0.04	0.77	27	EU-SILC versus ESS	− 0.03	0.86	27
EU-SILC versus EHIS	0.03	0.92	15	EU-SILC versus EHIS	0.25	0.19	15
EHIS versus ESS	− 0.01	1.00	14	EHIS versus ESS	0.18	0.38	14

Significance in bold (*p* < 0.05)

EHIS—European Health Interview Survey (2006/2009); ESS—European Social Survey (2008, 2010, 2012); EU-SILC—EU Statistics on Income and Living Conditions (2008, 2012) for 28 European countries (Finland, Sweden, Norway, Denmark, the UK, Ireland, the Netherlands, Belgium, Germany, Austria, Switzerland, France, Spain, Portugal, Italy, Greece, Cyprus, Slovenia, Croatia Czech Republic, Slovakia, Hungary, Poland, Bulgaria, Romania, Latvia, Lithuania, Estonia)

^a^ Countries without a pair are excluded from the rank comparison

^b^A value of − 1 indicates complete reversal between the two ranks being compared, 0 that the ranks are independent of each other, and 1 that they completely agree

## Discussion

### Summary of findings

EHIS estimates around 17% (men) and 23% (women) higher average prevalence of GALI disability than EU-SILC; 24% (men) and 29% (women) than ESS, whereas prevalence is not statistically significantly different between EU-SILC and ESS. The analyses stratified by education show that ESS estimates lower prevalence relative to EU-SILC only for the low educated; and that EHIS estimates higher prevalence across all educational groups, but more marked for the high educated than for the low educated. There is no agreement between surveys in ranking of countries by average prevalence.

On average, EU-SILC estimates the highest educational inequalities in GALI disability (ARR = 1.93 for men; ARR = 1.73 for women), followed by EHIS (ARR = 1.61 for men; ARR = 1.57 for women) and ESS (ARR = 1.57 for men; ARR = 1.55 for women). Educational inequalities are statistically significantly different between surveys for several countries.

There is no agreement between surveys in ranking of countries by educational inequalities in GALI prevalence, with the exception of a small positive correlation between EHIS and ESS for men (Kendall’s Tau = 0.40).

We observe a statistically significant association of GALI disability with collection mode of the survey, with remote interviewer (CATI) associated with lower GALI prevalence relative to present interviewer (PAPI and CAPI). However, the inclusion of survey characteristics does not account for the observed differences between surveys in prevalence or inequalities.

### Strengths and weaknesses

This is the first systematic analysis of the agreement of 3 European surveys in their estimates of GALI prevalence and educational inequalities. Unlike previous studies, we used micro-level data to explore variations both in relative (ARRs) and absolute (ARDs) terms. This is desirable considering that odds ratios (ORs) tend to be artificially high in the case of non-rare conditions (Tajeu et al. 2012) and that risk ratios are preferred over ORs as measures in epidemiologic studies. Additionally, we have used a structured framework to compare country-specific and average differences in GALI prevalence and inequalities as well as their association with survey characteristics, and have explicitly compared country rankings for these outcomes.

Limitations of the study include our inability to study the effect of differences in phrasing of the GALI question. We could not include GALI question differences in the pooled analyses with all surveys because there was no variation in GALI phrasing within EHIS (uses GALI standard phrasing throughout) and ESS (omits time reference throughout). We examined whether GALI phrasing significantly explained variation in GALI disability within EU-SILC, but we were unable to detect a statistically significant association (Online Resource 6). Omission of dimensions of the GALI question (being limited, in activities people usually do, because of health problems, for at least the past 6 months), as well as changes in wording and separation of the dimensions into several questions, has been shown to have an important effect on how individuals respond to self-reported questions (Cambois et al. 2016a; EHLEIS 2011; McClendon and O’Brien 1988).

### Interpretation of findings and comparison with previous studies

There are important differences in the prevalence and the educational inequalities of GALI disability between the surveys included in the analysis. These differences have not been explained by the survey characteristics included in our models. There are other factors that are hard to capture that could explain the observed differences in prevalence and inequalities of GALI disability. For instance, the nature of the surveys is different from one another. ESS has extensive information on beliefs, attitudes and behaviors of Europeans, while EHIS is rich in health-related questions and EU-SILC focuses more on socioeconomic and income variables. This means that the context of the survey where the GALI question is being asked varies across surveys, with respondents being primed with other types of questions that could alter their response to the GALI question. The context in which the survey takes place, the wording and format of the question and even adjacent questions have been shown to matter in the responses individuals provide to self-reports (Schwarz 1999).

Although survey characteristics did not significantly explain the reported differences, we found a significant association between prevalence of GALI disability and mode of data collection. The results indicate that surveys conducted by a remote interviewer (CATI) are associated with lower prevalence when compared to present interviewers (PAPI, CAPI). Prior research has shown that collection mode has an impact on data quality (Bowling 2005), as well as on response rate: Response rates are higher in face-to-face interviews (Demarest et al. 2013), and lower in telephone interviews (Sykes and Collins 1988).

Our results are consistent with previous studies that used SRH. Croezen et al. find that prevalence of SRH is significantly different between the three surveys they compare and find associations with several survey characteristics (response rate, sample size, collection mode). Toch-Marquardt (2017) finds something similar for prevalence of SRH and for occupational inequalities, and finds no consistency in regional patterns. The choice of survey has a major impact on the conclusions we draw both about prevalence and health inequalities. This is the case when looking at both educational and occupational inequalities in health. Furthermore, our analyses of the ranking of countries by survey also indicate that the conclusions we draw of best and worst performers are also affected by the choice of survey. This has important implications for the monitoring of health and cross-national comparisons.

Further research is necessary to identify factors that explain the differences between surveys. One promising approach is to exploit changes in the implementation of a survey (e.g., collection mode, sampling design, phasing of the question) within a given country to establish how these affect the measurement of important health indicators. As more years of data become available, changes in survey implementation toward harmonization will provide opportunities to better understand the (lack of) agreement of health measurements across surveys.

The implications of these findings for health monitoring are important. At the national level, it is difficult to make reliable assessments of the prevalence of disability since the agreement between different surveys is lacking and there is no gold standard among the three surveys. This is also the case for the educational inequalities. For monitoring purposes at the country level, it is perhaps best to look at the trends over time for GALI disability and inequalities, and assess if the surveys agree in the trends. This would inform whether a country is consistently improving or worsening. International comparisons are even harder to perform reliably. Which countries are best and worst performers in terms of prevalence or inequalities in limitations depends strongly on the survey. As long as we have no way of knowing which survey represents reality, our only option is to combine all available data sources and search for patterns that are consistent between surveys. Although prevalences do not agree in magnitude, all three surveys estimate a higher prevalence for Latvia and Slovenia, and a lower prevalence for Cyprus. For educational inequalities, both ESS and EU-SILC estimate high risk ratios for Norway for men and Portugal and Slovenia for women.

Although we were unable to detect statistically significant effects of sample size and response rate, it is objectively desirable that both are optimized given financial constraints. This increases the accuracy of population health measurements and minimizes the risk of bias. From this perspective, it is perhaps legitimate to put more confidence in larger sample surveys with higher response rates like EU-SILC or EHIS. ESS has smaller sample sizes which also complicates working at subpopulation levels (Robine et al. 2003), particularly when stratifying by country, gender and education. However, ESS has other advantages, like a higher degree of ex ante harmonization than EU-SILC, whereas EHIS is conducted infrequently.

It is still unclear whether population levels of disability can be reliably measured with self-reports. The lack of agreement in prevalence and inequalities in disability and self-reported health calls for caution when using these surveys for cross-country comparisons.

### Conclusions and recommendations

We find that both prevalence and educational inequalities of GALI disability are significantly affected by the choice of survey. We arrive to different interpretations of the health status of a country, its relative position to other countries and the size of educational inequalities depending on what survey is used for the measurement. This has important implications for population health monitoring, as well as developing valid comparisons across countries.

Our findings add to existing literature that investigated the comparability of SRH and has determined that this self-reported measure also varies significantly across other widely used European surveys. Further study is necessary to elucidate the causes of these discrepancies, and further harmonization of wording of the GALI question is necessary. Meanwhile, one should be very cautious in using these surveys for cross-country comparisons of (inequalities in) GALI disability.

## Electronic supplementary material

Below is the link to the electronic supplementary material.

Supplementary material 1 (DOCX 315 kb)
