# Supplementary material for: Educational inequalities in Global Activity Limitation Indicator disability in 28 European Countries: Does the choice of survey matter?
Source: Int J Public Health. 2018 Nov 26;64(3):461–74. doi: 10.1007/s00038-018-1174-7 (PMC6451713; doi:10.1007/s00038-018-1174-7)
Supplement: Supplementary file 1 — Supplementary material 1 (DOCX 315 kb) [file 38_2018_1174_MOESM1_ESM.docx]

**International Journal of Public Health**

**Educational inequalities in Global Activity Limitation Indicator disability in 28 European Countries. Does the choice of survey matter?**

**Supplementary Files**

**Online Resource 1**

| **Table S1-1. Survey characteristic distribution - Based on country/survey data (ages 30-79) for 28 European Countries** | | | | | | |
| --- | --- | --- | --- | --- | --- | --- |
| **Characteristic/ Survey** | **European Health Interview Survey**  **(EHIS)** | **European Social Survey**  **(ESS)** | | | **EU-Survey Income Living Conditions**  **(EU-SILC)** | |
|  | **2006/08** | **2008** | **2010** | **2012** | **2008** | **2012** |
| **Sample size** |  |  |  |  |  |  |
| <2000 | 1 | 15 | 16 | 13 | 0 | 0 |
| 2001 to 5000 | 3 | 12 | 9 | 10 | 0 | 0 |
| 5001 to 10.000 | 7 | 0 | 0 | 0 | 2 | 1 |
| 10.001 to 15.000 | 0 | 0 | 0 | 0 | 12 | 15 |
| 15.001 to 20.000 | 2 | 0 | 0 | 0 | 4 | 4 |
| >20.000 | 3 | 0 | 0 | 0 | 8 | 8 |
| *Total* | 16 | 27 | 25 | 23 | 26 | 28 |
| **Individual Response Rate** | | | | | | |
| <50% | 0 | 5 | 4 | 4 | 0 | 0 |
| 50-60% | 2 | 8 | 10 | 8 | 0 | 0 |
| 60-70% | 2 | 8 | 7 | 5 | 5 | 2 |
| 70-80% | 4 | 6 | 3 | 6 | 8 | 5 |
| >80% | 3 | 0 | 1 | 0 | 12 | 6 |
| *Total* | 11 | 27 | 25 | 23 | 25 | 13 |
| **Sampling Design** |  |  |  |  |  |  |
| Simple, random one/multi-stage | 0 | 4 | 5 | 6 | 1 | 0 |
| Stratified, random, one-multi stage | 15 | 22 | 19 | 16 | 13 | 13 |
| Stratified, systematic, one/multi-stage | 0 | 1 | 1 | 1 | 12 | 15 |
| *Total* | 15 | 27 | 25 | 23 | 26 | 28 |
| **Collection Mode** * | | | | | | |
| Present Interviewer (PAPI, CAPI) | 8 | 27 | 25 | 23 | 17 | 17 |
| Remote Interviewer (CATI) | 0 | 0 | 0 | 0 | 5 | 5 |
| Two types of data collection | 8 | 0 | 0 | 0 | 4 | 6 |
| *Total* | 16 | 27 | 25 | 23 | 26 | 28 |
| **Global Activity Limitation Indicator (GALI) Comparabilty^a^** | | | | | | |
| GALI Standard | 16 | 0 | 0 | 0 | 9 | 9 |
| No time reference | 0 | 27 | 25 | 23 | 1 | 1 |
| Personal reference | 0 | 0 | 0 | 0 | 7 | 7 |
| Unknown comparability | 0 | 0 | 0 | 0 | 6 | 5 |
| *Total* | 16 | 27 | 25 | 25 | 23 | 23 |
| **Proxy Allowed** | | | | | | |
| No | 5 | 27 | 25 | 23 | 7 | 9 |
| Yes | 9 | 0 | 0 | 0 | 19 | 19 |
| *Total* | 14 | 27 | 25 | 23 | 26 | 28 |
| ^a^ Information on the GALI question can be found in the ESS and EHIS quality reports. For EU-SILC it is available in the “Assesment of the GALI question used in SILC since 2008” from EHLEIS  Surveys included EHISw1(2006/09), ESS(2008,2010,2012) and EU-SILC (2008,2012) for 28 European countries.  *CAPI (Computer Assisted Personal Interview ); PAPI(Pencil and Paper Interview); CATI(Computer Assisted Telephone Interview) | | | | | | |

For EHIS, in the 11 countries where we have information on response rates, 7 have response rates higher than 70%, while the rest of countries have response rates between 50 and 70%. Around half of the countries included in ESS for a given year have response rates lower than 60%. The remaining countries have response rates between 60 and 80%. Finally, EU-SILC countries have higher response rates than the other surveys, with most countries above 70% and the remaining around 60-70%.

For sample size, in EHIS most countries have a sample size above 5,000 (7 countries) and some have larger sample sizes that exceed 15,000 (5 countries) . This contrasts with ESS, where most countries have sample sizes below 2,000 respondents (around 15 countries) and the remaining do not exceed 5,000 (around 10 countries). EU-SILC has the largest sample sizes, with only 1 country having sample size below 10,000; most countries between 10,000 and 20,000 (15 countries in 2012) and several with sample sizes over 20,000 (8 countries).

All of the countries in EHIS use a stratified, random, single or multi stage sampling design. Most of the countries in ESS also use this sampling design, with only 5 using simple, random, one/multi-stage sampling. Around half of the EU-SILC countries use a stratified, random, one/multi –stage sampling design. The other half uses a stratified, systematic, one/multi-stage approach, with only 1 country using simple, random, one/multi-stage design (Denmark).

Regarding collection mode of the surveys, all ESS countries use either PAPI (Paper Assisted Personal Interview) or CAPI (Computer Assisted Personal Interview), which have been combined in a “present interviewer” category in our study. In the case of EHIS, half of the countries collect their information solely through a present interviewer, while the other half combines collection methods. For EU-SILC, there is more variation in collection mode. Most countries use the present interviewer modes (17), while fewer use CATI (6 countries) – Computer Assisted Telephone Interview - and a similar number use two types plus self-administered (6 countries).

Important differences are found in the phrasing of the GALI question. All countries in ESS omit the time reference of the question. On the other end, EHIS uses the standard version of the GALI question for all countries. Displaying more variation, 9 EU-SILC countries use the standard GALI question, 1 country splits the question into two parts, where the second parts asks if the limitation lasted for more or less than 6 months(Netherlands), 7 countries provide a personal reference (i.e “in your daily activities”, whereas the standard is “activities people do”), while 6 are either make no reference to what type of daily activities (Austria, Portugal), no translation available (Spain, Lithuania, Romania) and the UK with a completely different GALI question that uses a filter question and partitions it in segments (9).

Use of proxy respondents also varies across surveys. ESS does not allow for substitution of respondents with proxy respondents. EHIS allows proxy use in 9 of the 14 countries we have information for, while the remaining 5 do not permit their use. Finally, 19 of the EU-SILC countries allow the use of proxy respondents and 9 do not.

| **Table S1-2. Sample Size & Individual Response Rate by Year - Based on country/survey data (ages 30-79) for 28 European Countries** | | | | | | | |
| --- | --- | --- | --- | --- | --- | --- | --- |
|  |  | EU- Statistics Income Living Conditions (EU-SILC) ^a^ | | European Social Survey (ESS)^b^ | | | E.Health Interview Survey (EHIS)^c^ |
| Code | Country | 2008 | 2012 | 2008 | 2010 | 2012 | 2006-08 |
| FI | Finland | 21,131  (81.77%) | 20,481  (79.80%) | 2.195  (68.44%) | 1,878  (59.45%) | 2,197  (67.27%) | - |
| SE | Sweden | 14,889  (74.05%) | 13,307  (n.a) | 1,830  (62.16%) | 1,497  (50.99%) | 1,847  (52.44%) | - |
| NO | Norway | 10.897  (62.70%) | 12,177  (n.a) | 1.370  (60.44%) | 1,548  (58.04%) | 1,624  (54.92%) | - |
| DK | Denmark | 11,545  (55.27%) | 10.868  (n.a) | 1,613  (53.88%) | 2,900  (55.4%) | 1,650  (49.43%) | - |
| UK | United Kingdom | 16,825  (72.98%) | 18,336  (62.79%) | 2,352  (55.77%) | 2.422  (56.3%) | 2,286  (53.06%) | - |
| IE | Ireland | 10,116  (76.25%) | 8,799  (n.a) | 1,764  (51.55%) | 2,576  (65.17%) | 2,628  (67.94%) | - |
| NL | Netherlands | 19,519  (81.66%) | 19,529  (n.a) | 1,778  (49.83%) | 1,829  (60.03%) | 1,845  (55.09%) | - |
| BE | Belgium | 12,154  (64.33%) | 11,192  (61.84%) | 1,760  (58.86%) | 1,704  (53.43%) | 1,869  (58.74% | 9,330  (n.a) |
| DE | Germany | 24,336  (75.22%) | 23,587  (n.a) | 2,751  (47.99%) | 3,031  (30.52%) | 2,958  (33.76%) | - |
| AT | Austria | 10,995  (70.77%) | 11,477  (76.62%) | - | - | - | 15,474  (63.1%) |
| CH | Switzerland | 13,230  (n.a) | 14,384  (n.a) | 1,484  (45.7%) | 1,506  (53.31%) | 1,493  (51.73%) | - |
| FR | France | 20,125  (82.45%) | 22,742  (82%) | 2,073  (49.38%) | 1,728  (47.06%) | 1,968  (62.05%) | 24,689  (n.a) |
| ES | Spain | 30,082  (63.25%) | 28,210  (77.18%) | 2,570  (66.79%) | 1,885  (68.52%) | 1,889  (70.28%) | 22,188  (n.a) |
| PT | Portugal | 10,101  (90.97%) | 13,584  (n.a) | 2,367  (75.74%) | 2,150  (67.08%) | 2,151  (77.12%) | - |
| IT | Italy | 44,286  (84.80%) | 40,287  (70.63%) | - | - | 960  (36.04%) | - |
| EL | Greece | 14,123  (89.43%) | 11,698  (n.a) | 2,072  (74.27%) | 2,715  (65.6%) | - | 6,172  (n.a) |
| CY | Cyprus | 8,090  (91.16%) | 11.044  (93.62%) | 1,215  (78.74%) | 1,083  (69.47%) | 1,116  (76.75%) | 6,931  (81.6%) |
| SI | Slovenia | 25,005  (76.05%) | 24,003  (76.58%) | 1,286  (59.1%) | 1,403  (63.39%) | 1,257  (57.74%) | 2,118  (n.a) |
| HR | Croatia | - | 13,228  (n.a) | 1,819  (49.88) | 1,649  (54.49%) | - | - |
| CZ | Czech Republic | 22,754  (80.82%) | 17,310  (82.30%) | 2,018  (69.49%) | 2,387  (70.16%) | 2,009  (68.4%) | 1,955  (56%) |
| SK | Slovakia | 14,098  (92.25%) | 13,602  (n.a) | 1,810  (72.55%) | 1,856  (74.66%) | 1,847  (74.09%) | 4,972  (66%) |
| HU | Hungary | 18.710  (80.44%) | 23,846  (n.a) | 1,544  (61.29%) | 1,561  (49.15%) | 2,014  (64.53%) | 5,051  (80.6%) |
| PL | Poland | 33,801  (75.51%) | 30,755  (n.a) | 1,619  (71.2%) | 1,751  (70.26%) | 1,898  (74.87%) | 35,100  (72%) |
| BG | Bulgaria | 10,373  (66.07%) | 12,886  (83.19%) | 2,230  (74.98%) | 2,434  (81.43%) | 2,260  (74.74%) | 5,661  (73.8%) |
| RO | Romania | 16,527  (94.98%) | 15,856  (94.91%) | 2,146  (67.95%) | - | - | 18,172  (89%) |
| LV | Latvia | 10,910  (75.27%) | 12,964  (n.a) | 1,980  (57.88%) | - | - | 6,458  (72%) |
| LT | Lithuania | 10,473  (83.41%) | 11,224  (85.83%) | - | 1,677  (39.41%) | 2,109  (49.61%) | - |
| EE | Estonia | 10,851  (78.32%) | 11.902  (n.a) | 1,662  (57.38%) | 1,800  (56.21%) | 2,380  (67.83%) | 6,429  (58.4%) |
| Total | | 27 | 28 | 25 | 24 | 23 | 15 |
| ^a^ Obtained from 2008 Comparative EU Final Quality Report, individual country reports and the Metadata for Official Statistics of the German Microdata Lab (Leibniz Institute for the Social Sciences).  ^b^ Obtained from the ESS4 and ESS5 Documentation Reports  ^c^ Obtained from the “Synthesis report on use of EHIS quality assessment criteria – Final Report” (Gauci, 2011 | | | | | | | |

| **Table S1-3. Collection Mode byYear^*^  - Based on country/survey pooled data (ages 30-79) for 28 European Countries** | | | | | | | | |
| --- | --- | --- | --- | --- | --- | --- | --- | --- |
|  |  | EU- Statistics Income Living Conditions (EU-SILC) ^a^ | | European Social Survey (ESS)^b^ | | | E.Health Interview Survey (EHIS)^c^ | |
| Code | Country | 2008 | 2012 | 2008 | 2010 | 2012 | 2006-08 | |
| FI | Finland | CATI | CATI | CAPI | CAPI | CAPI | - | |
| SE | Sweden | CATI | CATI | CAPI | CAPI | CAPI | - | |
| NO | Norway | CATI | CATI | CAPI | CAPI | CAPI | - | |
| DK | Denmark | CATI | CATI, Self-admin | CAPI | CAPI | CAPI | - | |
| UK | United Kingdom | CAPI | CAPI | CAPI | CAPI | CAPI | - | |
| IE | Ireland | CAPI | CAPI | CAPI | CAPI | CAPI | - | |
| NL | Netherlands | CATI | CATI | CAPI | CAPI | CAPI | - | |
| BE | Belgium | CAPI | CAPI | CAPI | CAPI | CAPI | PAPI, Self-admin | |
| DE | Germany | Self-admin | Self-admin | CAPI | CAPI | CAPI | - | |
| AT | Austria | CAPI, CATI | CAPI, CATI | - | - | - | CAPI | |
| CH | Switzerland | CAPI, CATI | CAPI, CATI | PAPI | PAPI | PAPI | - | |
| FR | France | CAPI | CAPI | CAPI | CAPI | CAPI | CAPI, Self-admin | |
| ES | Spain | CAPI | CAPI | CAPI | CAPI | CAPI | CAPI | |
| PT | Portugal | CAPI | CAPI | CAPI | CAPI | CAPI | - | |
| IT | Italy | PAPI | CAPI | - | - | PAPI | - | |
| EL | Greece | PAPI | PAPI | PAPI | PAPI | - | PAPI | |
| CY | Cyprus | CAPI | CAPI | PAPI | PAPI | PAPI | CAPI | |
| SI | Slovenia | CAPI, CATI | CAPI, CATI | PAPI | PAPI | PAPI | PAPI, Self-admin | |
| HR | Croatia | - | CAPI | CAPI | CAPI | - | - | |
| CZ | Czech Republic | PAPI | CAPI, PAPI | PAPI | PAPI | PAPI | PAPI | |
| SK | Slovakia | PAPI | PAPI | PAPI | PAPI | PAPI | PAPI, Self-admin | |
| HU | Hungary | PAPI | PAPI | PAPI | PAPI | CAPI | PAPI | |
| PL | Poland | PAPI | PAPI | PAPI | PAPI | PAPI | PAPI | |
| BG | Bulgaria | PAPI | PAPI | PAPI | PAPI | PAPI | PAPI, Self-admin | |
| RO | Romania | PAPI | PAPI | PAPI | - | - | PAPI, Self-admin | |
| LV | Latvia | CAPI, CATI | CAPI, CATI | PAPI | - | - | CAPI, PAPI | |
| LT | Lithuania | PAPI, CATI | PAPI, CATI | - | PAPI | PAPI | - | |
| EE | Estonia | CAPI | CAPI | PAPI | PAPI | PAPI | PAPI | |
|  | | | | | | | | |
| Total | | 27 | 28 | 25 | 24 | 23 | | 15 |
| ^*^ Collection mode is included in the table only if the % of the sample collected surpasses 15% of the total.  ^a^ Obtained from 2008 Comparative EU Final Quality Report, individual country reports and the Metadata for Official Statistics of the German Microdata Lab (Leibniz Institute for the Social Sciences).  ^b^ Obtained from the ESS4 and ESS5 Documentation Reports  ^c^ Obtained from the “Synthesis report on use of EHIS quality assessment criteria – Final Report” (Gauci, 2011)  Surveys included EHISw1(2006/09), ESS(2008,2010,2012) and EU-SILC (2008,2012) for 28 European countries. | | | | | | | | |

| **Table S1-4. Sampling Design^d^ by Survey, Country and Year^*-^ Based on country/survey pooled data (ages 30-79) for 28 European Countries** | | | | | | | |
| --- | --- | --- | --- | --- | --- | --- | --- |
|  |  | EU- Statistics Income Living Conditions (EU-SILC) ^a^ | | European Social Survey (ESS)^b^ | | | E.Health Interview Survey (EHIS)^c^ |
| Code | Country | 2008 | 2012 | 2008 | 2010 | 2012 | 2006-08 |
| FI | Finland | systematic, stratified, m | systematic, stratified, m | random, simple, 1 | random, simple, 1 | random, simple, 1 | - |
| SE | Sweden | systematic, stratified, 1 | n.a | random, stratified, 1 | random, stratified, 1 | random, stratified, 1 | - |
| NO | Norway | random, stratified, 1 | random, stratified, 1 | random, simple, 1 | random, simple, 1 | random, simple, 1 | - |
| DK | Denmark | random, simple, 1 | n.a | random, simple, 1 | random, simple, 1 | random, simple, 1 | - |
| UK | United Kingdom | systematic, stratified, m | systematic, stratified, m | random, stratified, m | random, stratified, m | random, stratified, m | - |
| IE | Ireland | random, stratified, m | n.a | random, stratified, m | random, stratified, m | random, stratified, m | - |
| NL | Netherlands | systematic, stratified, m | n.a | random, stratified, m | random, stratified, m | random, stratified, m | - |
| BE | Belgium | systematic, stratified, m | n.a | random, stratified, m | random, stratified, m | random, stratified, m | random, stratified, m |
| DE | Germany | random, stratified, 1 | n.a | random, stratified, m | random, stratified, m | random, stratified, m | - |
| AT | Austria | random, stratified, 1 | random, stratified, 1 | - | - | - | random, stratified, 1 |
| CH | Switzerland | proportional, stratified, m | proportional, stratified, m | random, stratified, m | random, stratified, m | random, stratified, m | - |
| FR | France | random, stratified, m | n.a | random, stratified, m | random, stratified, m | random, stratified, m | random, stratified, m |
| ES | Spain | systematic, stratified, m | systematic, stratified, m | random, stratified, m | random, stratified, m | random, stratified, m | random, stratified, m |
| PT | Portugal | systematic, stratified, m | systematic, stratified, m | random, stratified, m | random, stratified, m | random, stratified, m | - |
| IT | Italy | systematic, stratified, m | systematic, stratified, m | - | - | random, stratified, m | - |
| EL | Greece | systematic, stratified, m | n.a | random, stratified, m | random, stratified, m | - | random, stratified, m |
| CY | Cyprus | random, stratified, 1 | random, stratified, 1 | random, stratified, m | random, stratified, m | random, stratified, m | random, stratified, 1 |
| SI | Slovenia | systematic, stratified, m | systematic, stratified, m | random, stratified, m | random, stratified, m | random, stratified, m | random, stratified, m |
| HR | Croatia | - | n.a | random, stratified, m | random, stratified, m | - | - |
| CZ | Czech Republic | random, stratified, m | random, stratified, m | random, stratified, m | random, stratified, m | random, stratified, m | random, stratified, m |
| SK | Slovakia | random, stratified, 1 | random, stratified, 1 | random, stratified, m | random, stratified, m | random, stratified, m | random, stratified, m |
| HU | Hungary | random, stratified, m | random, stratified, m | random, stratified, m | random, stratified, m | random, stratified, m | random, stratified, m |
| PL | Poland | random, stratified, m | random, stratified, m | random, stratified, m | random, stratified, m | random, stratified, m | random, stratified, m |
| BG | Bulgaria | systematic, stratified, m | systematic, stratified, m | random, stratified, m | random, stratified, m | random, stratified, m | random, stratified, m |
| RO | Romania | random, stratified, m | random, stratified, m | random, stratified, m | - | - | random, stratified, m |
| LV | Latvia | systematic, stratified, m | systematic, stratified, m | random, stratified, m | - | - | random, stratified, m |
| LT | Lithuania | random, stratified, 1 | random, stratified, 1 | - | random, stratified, m | random, stratified, m | - |
| EE | Estonia | systematic, stratified, 1 | systematic, stratified, 1 | systematic, stratified, 1 | systematic, stratified, 1 | random, stratified, m | systematic, stratified, 1 |
| Total | | 27 | 28 | 25 | 24 | 23 | 15 |
| Surveys included EHISw1(2006/09), ESS(2008,2010,2012) and EU-SILC (2008,2012) for 28 European countries. | | | | | | | |

| **Table S1-5. Use of Proxy Respondents Allowed Year^*^ -Based on country/survey pooled data (ages 30-79) for 28 European Countries** | | | | |
| --- | --- | --- | --- | --- |
|  |  | EU- Statistics Income Living Conditions  (EU-SILC) ^a^ | European Social Survey (ESS)^b^ | E.Health Interview Survey (EHIS)^c^ |
| Code | Country |  |  |  |
| FI | Finland | No | No | - |
| SE | Sweden | No | No | - |
| NO | Norway | No | No | - |
| DK | Denmark | Yes | No | - |
| UK | United Kingdom | No | No | - |
| IE | Ireland | Yes | No | - |
| NL | Netherlands | No | No | - |
| BE | Belgium | Yes | No | Yes |
| DE | Germany | Yes | No | - |
| AT | Austria | Yes | No | Yes |
| CH | Switzerland | Yes | No | - |
| FR | France | Yes | No | Yes |
| ES | Spain | Yes | No | Yes |
| PT | Portugal | Yes | No | - |
| IT | Italy | Yes | No | - |
| EL | Greece | Yes | No | Yes |
| CY | Cyprus | Yes | No | Yes |
| SI | Slovenia | No | No | No |
| HR | Croatia | Yes | No | - |
| CZ | Czech Republic | Yes | No | No |
| SK | Slovakia | No | No | No |
| HU | Hungary | No | No | No |
| PL | Poland | Yes | No | Yes |
| BG | Bulgaria | Yes | No | Yes |
| RO | Romania | Yes | No | Yes |
| LV | Latvia | Yes | No | Yes |
| LT | Lithuania | Yes | No | - |
| EE | Estonia | No | No | No |
| ^a^ Obtained from 2008 Comparative EU Final Quality Report, individual country reports and the Metadata for Official Statistics of the German Microdata Lab (Leibniz Institute for the Social Sciences).  ^b^ Obtained from the ESS4 and ESS5 Documentation Reports  ^c^ Obtained from the “Synthesis report on use of EHIS quality assessment criteria – Final Report” (Gauci, 2011)  Surveys included EHISw1(2006/09), ESS(2008,2010,2012) and EU-SILC (2008,2012) for 28 European countries. | | | | |

| **Table S2-1Adjusted Risk Difference of Global Activity Limitation Indicator disability for survey (ref=0, European Union Statistics on Income and Living Conditions ) and survey characteristics - Based on country/survey pooled data (ages 30-79) for 28 European countries** | | | | | | | | | | | | | |
| --- | --- | --- | --- | --- | --- | --- | --- | --- | --- | --- | --- | --- | --- |
| **Men** | | **Model 1** | | **Model 2** | | **Model 3** | | **Model 4** | | **Model 5** | | **Model 6** | |
| **Survey** | | ARD | 95% CI | ARD | 95% CI | ARD | 95% CI | ARD | 95% CI | ARD | 95% CI | ARD | 95% CI |
| EU-SILC | | 0 | - | 0 | - | 0 | - | 0 | - | 0 | - | 0 | - |
| EHIS | | **4.23** | [2.39, 6.06] | **5.44** | [2.46, 7.70] | **3.76** | [2.11, 5.42] | **3.91** | [1.95, 5.87] | **4.58** | [2.61, 6.55] | **7.28** | [1.67, 12.89] |
| ESS | | -1.43 | [-3.99, 1.13] | -1.90 | [-4.95, 8.15] | -3.74 | [-7.8,0.01] | -2.36 | [-4.95, 0.24] | -3.75 | [-7.8,0.0] | -0.00 | [-4.74, 4.57] |
| **Education** |  | | |  | |  | |  | |  | |  | |
| Low | | **13.36** | [12.28, 14.44] | **13.22** | [12.17, 15.46] | **13.30** | [12.22-14.37] | **13.33** | [13.92, 15.97] | **13.32** | [12.26, 14.38] | **13.33** | [12.22-14.37] |
| Medium | | **6.10** | [4.94, 7.25] | **5.99** | [4.70, 7.55] | **6.11** | [4.96, 7.26] | **6.06** | [4.92, 7.19] | **6.05** | [4.93, 7.17] | **6.13** | [4.96, 7.26] |
| High | | 0 | - | 0 | - | 0 | - | 0 | - | 0 | - | 0 | - |
| **Response rate** | |  |  | 0.00 | [-0.01, 0.01] |  |  |  |  |  |  |  |  |
| **Sample Size (x1000)** | |  |  |  |  | -0.001 | [-0.01, 0.01] |  |  |  |  |  |  |
| **Sampling Design** | | | |  | |  | |  | |  | |  | |
| Simple, random | |  |  |  |  |  |  | 0 | - |  |  |  |  |
| Stratified, random | |  |  |  |  |  |  | -5.02 | [-11.65, 1.60] |  |  |  |  |
| Stratified, systematic | |  |  |  |  |  |  | -5.29 | [-12.60, 1.68] |  |  |  |  |
| **Collection Mode** | | | | | |  | |  | |  | |  | |
| CAPI and PAPI | |  |  |  |  |  |  |  |  | 0 | - |  |  |
| CATI | |  |  |  |  |  |  |  |  | **-4.77** | [-9.04, -0.50] |  |  |
| Other | |  |  |  |  |  |  |  |  | -2.78 | [-3.44, 8.78] |  |  |
| **Proxy Allowed** | | | | | | | |  | |  | |  | |
| No | |  |  |  |  |  |  |  |  |  |  | 0 | - |
| Yes | |  |  |  |  |  |  |  |  |  |  | 2.65 | [-2.52, 7.81] |
| N | | 387,011 | | 295,064 | | 387,011 | | 384,740 | | 387,011 | | 387,228 | |
| Wald test (p-val) | | - | | 0.76 | | 0.07 | | 0.29 | | **0.00** | | 0.31 | |
| **Women** | | **Model 1** | | **Model 2** | | **Model 3** | | **Model 4** | | **Model 5** | | **Model 6** | |
| **Survey** | | ARD | 95% CI | ARD | 95% CI | ARD | 95% CI | ARD | 95% CI | ARD | 95% CI | ARD | 95% CI |
| EU-SILC | | 0 | - | 0 | - | 0 | - | 0 | - | 0 | - | 0 | - |
| EHIS | | **6.60** | [4.63, 8.56] | **6.68** | [3.42, 9.94] | **6.20** | [4.48, 7.91] | **6.10** | [3.91, 8.29] | **7.37** | [4.86, 9.87 ] | **6.68** | [4.72, 8.64] |
| ESS | | -1.29 | [-3.99, 1.13] | -2.40 | [-5.84, 1.04] | -3.25 | [-8.45, 1.95] | -1.76 | [-5.60, 2.08] | -3.29 | [-6.45, -0.14] | -3.17 | [-7.37, 1.03] |
| **Education** | | | | | | | | | |  | |  | |
| Low | | **13.94** | [12.28, 14.44] | **13.59** | [12.36, 14.82] | **13.90** | [13.38, 16.13] | **13.85** | [12.66, 15.02] | **13.86** | [12.65, 15.04] | **13.93** | [12.70, 15.17] |
| Medium | | **5.53** | [4.79, 6.26] | **5.49** | [4.67, 6.31] | **5.53** | [4.79, 6.26] | **5.48** | [4.75, 6.20] | **5.53** | [4.81, 6.26] | **5.53** | [4.80, 6.26] |
| High | | 0 | - | 0 | - | 0 | - | 0 | - | 0 | - | 0 | - |
| **Response rate** | |  |  | 0.00 | [-0.01, 0.01] |  |  |  |  |  |  |  |  |
| **Sample Size (x1000)** | |  |  |  |  | 0.00 | [-0.004, 0.009] |  |  |  |  |  |  |
| **Sampling Design** | | | | | | | | | | | |  | |
| Simple, random | |  |  |  |  |  |  | 0 | - |  |  |  |  |
| Stratified, random | |  |  |  |  |  |  | -2.58 | [-8.42, 3.25] |  |  |  |  |
| Stratified, systematic | |  |  |  |  |  |  | -2.89 | [-9.32, 3.54] |  |  |  |  |
| **Collection Mode** | | | | | | | | | | | | | |
| CAPI and PAPI | |  |  |  |  |  |  |  |  | 0 | - |  |  |
| CATI | |  |  |  |  |  |  |  |  | **-4.96** | [-9.9, 0.01] |  |  |
| Other | |  |  |  |  |  |  |  |  | -3.70 | [-7.05, 0.35] |  |  |
| **Proxy Allowed** | | | | | | | | | | | | | |
| No | |  |  |  |  |  |  |  |  |  |  | 0 | - |
| Yes | |  |  |  |  |  |  |  |  |  |  | -3.56 | [-9.02, 1.90] |
| N | | 445,538 | | 337,695 | | 445,538 | | 442,953 | | 445,538 | | 445,538 | |
| Wald test (p-val) | | - | | 0.45 | | 0.20 | | 0.65 | | **0.03** | | 0.20 | |

**
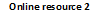
**Model 1 includes all pooled data for countries and surveys, stratified only by sex. The model is ${logit(GALI}_{iksce})=\beta_{0}+ \beta_{k}Age+ \beta_{s}{Survey}_{s}+ {Country}_{c}$ $+ \beta_{e}{Education}_{e}$

Models 2-6 use $logit({GALI}_{ikesc})= \beta_{k}Age+ \beta_{s}{Survey}_{s}+ \beta_{e}{Education}_{e}+\beta_{c}{Country}_{c}$ + $\beta_{n}{SurveyChar}_{n}$ . The Wald tests compare baseline Model 1 with Model 2-6.

*CAPI (Computer Assisted Personal Interview ); PAPI(Pencil and Paper Interview); CATI(Computer Assisted Telephone Interview)

EHIS – European Health Interview Survey (2006/09); ESS - European Social Survey (2008,2010,2012) ; EU-SILC- EU Statistics on Income and Living Conditions (2008,2012)

| **Table S2-2. Adjusted Risk Difference of disability prevalence (between surveys) for the pooled dataset, stratified by education for men and women (ages 30-79) - Based on country/survey pooled data (ages 30-79) for 28 European Countries** | | | | | | |
| --- | --- | --- | --- | --- | --- | --- |
| **Men** | | | | | | |
| ***Survey*** | ***Model A***  ***Low Educated*** | | ***Model B***  ***Medium Educated*** | | ***Model C***  ***High Educated*** | |
| EU-Survey Income Living Conditions (EU-SILC) | 0.00 | ref | 0.00 | ref | 0.00 | ref |
| European Health Interview Survey (EHIS) | **2.54** | [0.20, 15.61] | **5.70** | [2.31, 12.31] | **4.84** | [2.92, 6.76] |
| European Social Survey (ESS) | **-3.80** | [-6.62, -0.99] | -0.10 | [-4.12, 2.42] | 1.20 | [-0.10, 3.21] |
| n | 119,290 | | 180,158 | | 87,563 | |
| **Women** | | | | | | |
| EU-Survey Income Living Conditions (EU-SILC) | 0.00 | ref | 0.00 | ref | 0.00 | ref |
| European Health Interview Survey (EHIS) | **6.43** | [4.12, 8.74] | **7.13** | [4.77, 9.49] | **6.09** | [3.35, 8.83] |
| European Social Survey (ESS) | **-3.48** | [-6.88, -0.01] | -0.01 | [-4.20, 2.92] | 1.07 | [2.02, 4.15] |
| n | 156,327 | | 190,606 | | 98,505 | |
| **Significance at the 5% level in bold**  Models are stratified by sex and education. The model corresponds to ${logit(GALI}_{iksc})=\beta_{k}Age+ \beta_{s}{Survey}_{s}+\beta_{c}{Country}_{c}$ . Errors are clustered at the country level.  EHIS – European Health Interview Survey (2006/09); ESS - European Social Survey (2008,2010,2012) ; EU-SILC- EU Statistics on Income and Living Conditions (2008,2012) .  Absolute education differences are based on the same logistic regression models as relative differences. The STATA command *adjrr* also provides with estimates of adjusted risk differences (ARD) i n addition to the adjusted risk ratios (ARR) presented in the main analysis.  A stratified analysis by education indicates that ESS estimate a significantly lower prevalence (-3.80 percentage points for men and -3.48 percentage points for women) of GALI disability than EU-SILC for the low educated group, but not for the medium and high educated. | | | | | | |

| **Table S2-3. Educational Inequalities (low vs high) in disability prevalence (adjusted risk difference - ARD and 95% Confidence Intervals) by survey - Based on country/survey pooled data (ages 30-79) for 28 European countries ^a^** | | | | | | | | | | | | |
| --- | --- | --- | --- | --- | --- | --- | --- | --- | --- | --- | --- | --- |
| **Men** | **Model 1** | | **Model 2** | | **Model 3** | | **Model 4** | | **Model 5** | | **Model 6** | |
| **Survey** | ARD | 95% CI | ARD | 95% CI | ARD | 95% CI | ARD | 95% CI | ARD | 95% CI | ARD | 95% CI |
| E.Health InterviewS. | 13.14 | [10.82, 15.46] | 14.07 | [10.69, 17.46] | 15.20 | [10.90, 19.51] | 14.79 | [10.95, 18.63] | 14.72 | [11.35, 18.09] | 15.10 | [10.81, 19.39[ |
|  |  |  |  |  |  |  |  |  |  |  |  |  |
| E. Social Survey | **10.29** | [8.78, 11.80] | **9.57** | [7.86, 11.28] | **9.90** | [7.87, 11.92] | **9.40** | [7.91, 10,98] | **9.84** | [8.30, 11.38] | **10.13** | [9.42, 11.84] |
| EU-Surv. Income Living Cond. | 15.30 | [13.71, 16.88] | 15.64 | [14.22, 17.06] | 15.96 | [14.61, 17.31] | 16.21 | [15.00, 17.42] | 16.12 | [14.89, 17.35] | 15.91 | [14.64, 17.18] |
| **Response rate** |  |  | 1.00 | [0.99, 1.00] |  |  |  |  |  |  |  |  |
| **Sample Size (x1000)** |  |  |  |  | 1.00 | [0.98, 1.02] |  |  |  |  |  |  |
| **Sampling Design** | | | | | | | | | | | | |
| Simple, random |  |  |  |  |  |  | 1 | - |  |  |  |  |
| Stratified, random |  |  |  |  |  |  | -4.66 | [-11.14, 1.81] |  |  |  |  |
| Stratified, systematic |  |  |  |  |  |  | -7.20 | [-14.33, 0.01] |  |  |  |  |
| **Collection Mode** | | | | | | | | | | | | |
| CAPI and PAPI |  |  |  |  |  |  |  |  | 1 | - |  |  |
| CATI |  |  |  |  |  |  |  |  | **-4.38** | [-8.57, -0.01] |  |  |
| Other |  |  |  |  |  |  |  |  | 2.86 | [-3.14, 8.86] |  |  |
| **Proxy Allowed** | | | | | | | | | | | | |
| No |  |  |  |  |  |  |  |  |  |  | 1 | - |
| Yes |  |  |  |  |  |  |  |  |  |  | 2.28 | [-2.72, 7.29] |
| N | 387,228 | | 295,134 | | 387,228 | | 384,957 | | 387,228 | | 387,228 | |
| F- test (p-val) | - | | 0.78 | | 0.75 | | 0.11 | | **0.02** | | 0.37 | |
|  | | | | | | | | | | | | |
| **Women** | **Model 1** | | **Model 2** | | **Model 3** | | **Model 4** | | **Model 5** | | **Model 6** | |
| **Survey** | ARD | 95% CI | ARD | 95% CI | ARD | 95% CI | ARD | 95% CI | ARD | 95% CI | ARD | 95% CI |
| E.Health InterviewS. | 15.48 | [12.31, 18.65] | 16.36 | [11.73, 20.24] | 15.52 | [12.39, 18.65] | 13.50 | [13.29, 20.01] | 14.77 | [13.29, 20.01] | 14.38 | [10.34, 18.43] |
|  | **11.63** | [9.55, 13.72 | **11.31** | [9.18, 13.45] | **11.21** | [9.11, 13.31] | 11.72 | [6.59, 10.99] | 10.46 | [8.31, 12.61] | 12.12 | [9.32, 14.92] |
| E. Social Survey | 15.11 | [14.39, 16.58] | 14.81 | [13.07, 16.55] | 15.30 | [13.69, 17.10] | 15.46 | [13.75, 17.26] | 15.50 | [13.75, 17.26] | 14.87 | [12.09, 17.66] |
| **Response rate** |  |  | 0.00 | [-0.01, 0.01] |  |  |  |  |  |  |  |  |
| **Sample Size (x1000)** |  |  |  |  | 0.00 | [-0.01, 0.01] |  |  |  |  |  |  |
| **Sampling Design** | | | | | | | | | | | | |
| Simple, random |  |  |  |  |  |  | 1 |  |  |  |  |  |
| Stratified, random |  |  |  |  |  |  | -2.55 | [-9.76, 3.14] |  |  |  |  |
| Stratified, systematic |  |  |  |  |  |  | -3.12 | [-9.70, 3.46] |  |  |  |  |
| **Collection Mode** | | | | | | | | | | | | |
| CAPI and PAPI |  |  |  |  |  |  |  |  | 1 |  |  |  |
| CATI |  |  |  |  |  |  |  |  | **-5.53** | [-10, -0.01] |  |  |
| Other |  |  |  |  |  |  |  |  | -3.58 | [-6.79, 0.36] |  |  |
| **Proxy Allowed** | | | | | | | | | | | | |
| No |  |  |  |  |  |  |  |  |  |  | 1 |  |
| Yes |  |  |  |  |  |  |  |  |  |  | -3.60 | [-8.99, 1.79] |
| N | 445,438 | | 337,695 | | 445,438 | | 442,953 | | 445,438 | | 445,438 | |
| F- test (p-val) | - | | 0.45 | | 0.18 | | 0.08 | | **0.03** | | 0.15 | |

**Significance In bold (p<0.05)**

Model 1 includes all pooled data for countries and surveys, stratified only by sex. The model is ${logit(GALI}_{iksce})=\beta_{0}+ \beta_{k}Age+ \beta_{s}{Survey}_{s}+ {Country}_{c}$ $+ \beta_{e}{Education}_{e}$ $\beta_{se}{Survey}_{s}*{Education}_{e}$

Models 2-6 use $logit({GALI}_{ikesc})= \beta_{k}Age+ \beta_{s}{Survey}_{s}+ \beta_{e}{Education}_{e}+\beta_{c}{Country}_{c} +\beta_{se}{Survey}_{s}*{Education}_{e}$ + $\beta_{n}{SurveyChar}_{n}$ (6) + $\beta_{ne}{SurveyChar}_{n}*{Education}_{e}$ [8]The Wald tests compare baseline Model 1 with Model 2-6.

*CAPI (Computer Assisted Personal Interview ); PAPI(Pencil and Paper Interview); CATI(Computer Assisted Telephone Interview)

**Table S2-4. Kendal’s Tau Correlations and associated p-value comparing country rank agreement (risk difference- ARD) between surveys (European Health Interview Survey 2006-09; European Social Survey 2008, 2010, 2012; European Union Statistics on Income and Living conditions 2008, 2012) for prevalence and educational inequalities - Based on country/survey pooled data (ages 30-79) for 28 European countries**

|  | | | |
| --- | --- | --- | --- |
| **Men** | | | |
| *Pair of Surveys* | *Kendall’s Tau*  *(-1 to 1)* | *p-value* | *n^a^* |
| EU-SILC vs  ESS | -0.04 | 0.77 | 27 |
| EU-SILC vs EHIS | -0.27 | 0.16 | 15 |
| EHIS vs  ESS | 0.45 | **0.03** | 14 |
|  | | | |
| ***Women*** | | | |
| *Pair of Surveys* | *Kendall’s Tau*  *(-1 to 1)* | *p-value* | *n^a^* |
| EU-SILC vs ESS | 0.08 | 0.53 | 27 |
| EU-SILC vs EHIS | -0.18 | 0.37 | 15 |
| EHIS vs  ESS | 0.07 | 0.74 | 14 |
| **Significance at the 5% level in bold**  ^a^ Countries without a pair are excluded from the rank comparison  ^b^ A value of -1 indicates complete reversal between the two ranks being compared, 0 that the ranks are independent of each other, and 1 that they completely agree.  EHIS – European Health Interview Survey (2006/09); ESS - European Social Survey (2008,2010,2012) ; EU-SILC- EU Statistics on Income and Living Conditions (2008,2012) . | | | |

The educational inequalities rank comparison based on ARD shows similar results of agreement as the ARR rank, with a significant correlation between EHIS and ESS for men (Tau = 0.45)

| **Table S3-1 Age-Standardized disability prevalence (age 30-79) by gender and survey (European Health Interview Survey 2006-09; European Social Survey 2008, 2010, 2012; European Union Statistics on Income and Living conditions 2008, 2012) - Based on country/survey data (ages 30-79) for 28 European Countries** | | | | | | | | | | | | | | | | |
| --- | --- | --- | --- | --- | --- | --- | --- | --- | --- | --- | --- | --- | --- | --- | --- | --- |
|  | **Prevalence (%)^a^** | | | | | |  |  | | **Prevalence (%)^a^** | | | | | | |
|  | **EU-SILC** | **95% CI** | **EHIS** | **95% CI** | **ESS** | **95% CI** |  |  | | **EU-SILC** | **95% CI** | **EHIS** | | **95% CI** | **ESS** | **95% CI** |
| **Men** |  |  |  |  |  |  |  | **Women** |  | |  |  | |  |  |  |
| Finland | 29.07 | [28.72, 29.77] | - | - | 32.74 | [30.83, 34.66] |  | Finland | 32.43 | | [31.69, 33.16] | | - | - | 31.55 | [29.68, 33.43] |
| Sweden | 13.06 | [12.46, 13.70] | - | - | 25.05 | [23.08, 27.01] |  | Sweden | 18.63 | | [17.88, 19.38] | | - | - | 28.1 | [26.11, 30.10] |
| Norway | 11.8 | [11.13, 12.47] | - | - | 24.46 | [22.43, 26.49] |  | Norway | 18.34 | | [17.52, 19.17] | | - | - | 29.3 | [27.12, 31.39] |
| Denmark | 24.39 | [23.48, 25.31] | - | - | 23.22 | [21.26, 25.18] |  | Denmark | 29.09 | | [28.12, 30.06] | | - | - | 29.37 | [27.25, 31.49] |
| UK | 19.48 | [18,81, 20.15] | - | - | 23.93 | [22.29, 25.57] |  | UK | 21.64 | | [20.96, 22.32] | | - | - | 25.9 | [24.29, 27.56] |
| Ireland | 19.43 | [18.50, 20.36] | - | - | 18.14 | [16.63, 19.65] |  | Ireland | 19.85 | | [18.19, 20.79] | | - | - | 15.16 | [13.73, 16.58] |
| Netherlands | 23.4 | [22.74, 24.06] | - | - | 21.46 | [19.70, 23.22] |  | Netherlands | 31.4 | | [30.69, 32.11] | | - | - | 30.19 | [28.24, 32.14] |
| Belgium | 20.99 | [20.15, 21.83] | 18.9 | [17.51, 20.28] | 23.24 | [21.42, 25.07] |  | Belgium | 23.08 | | [22.23, 23.94] | | 24.06 | [22.61, 25.52] | 25.81 | [23.91, 27.71] |
| Germany | 32.79 | [32.04, 33.38] | - | - | 30.77 | [29.19, 32.25] |  | Germany | 33.02 | | [32.37, 33.67] | | - | - | 29.25 | [27.75, 30.74] |
| Austria | 28.4 | [27.45, 29.34] | 34.41 | [33.19, 35.63] | - | - |  | Austria | 28.13 | | [27.21, 29.04] | | 35.37 | [34.18, 36.57] | - | - |
| Switzerland | 21.26 | [17.54, 19.00] | - | - | 17.16 | [15.39, 18.94] |  | Switzerland | 23.69 | | [22.90, 24.48] | | - | - | 19.55 | [17.78, 21.32] |
| France | 21.77 | [21.14, 22.41] | 24.34 | [23.45, 25.23] | 23.49 | [21.66, 25.32] |  | France | 23.45 | | [22.82, 24.80] | | 27.01 | [26.13, 27.85] | 23.93 | [22.19, 25.67] |
| Spain | 20.32 | [19.79, 20.84] | 21.18 | [20.31, 22.06] | 11.94 | [10.64, 13,23] |  | Spain | 22.8 | | [22.28, 23.31] | | 28.26 | [27.31, 29.20] | 18.16 | [16.70, 19.62] |
| Portugal | 22.5 | [21.65, 23.38] | - | - | 13.75 | [12.36, 15.14] |  | Portugal | 29.33 | | [28.46, 30.19] | | - | - | 17.27 | [15.90, 18.64] |
| Italy | 23.19 | [22.76, 23.63]] | - | - | 16.18 | [12.41, 19.96] |  | Italy | 26.71 | | [26.27, 27.15] | | - | - | 17.7 | [14.00, 21.39] |
| Greece | 17.41 | [16.71, 18.11] | 17.02 | [15.57, 18.47] | 10.23 | [8.85, 11.62] |  | Greece | 20.16 | | [19.45, 20.87] | | 26.39 | [24.73, 28.00] | 16.01 | [14.52, 17.94] |
| Cyprus | 20.99 | [20.05, 21.94] | 18.44 | [16.93, 19.95] | 15.77 | [13.73, 17.80] |  | Cyprus | 22.66 | | [21.75, 23.57] | | 22.71 | [21.56, 24.26] | 21.94 | [19.85, 24.02] |
| Slovenia | 28.95 | [28.31, 29.54] | 35.97 | [32.72, 39.22] | 29.7 | [27.46, 31.93] |  | Slovenia | 31.14 | | [30.52, 31.72] | | 40.52 | [37.22, 43.82] | 29.5 | [27.29, 31.71] |
| Croatia | 21.34 | [20.24, 22.43] | - | - | 27.63 | [25.04, 30.23] |  | Croatia | 21.49 | | [20.24, 22.28] | | - | - | 24.03 | [21.83, 26.22] |
| Czech Rep. | 20.89 | [20.19, 21.60] | 29.7 | [26.19, 33.20] | 26.26 | [24.58, 27.94] |  | Czech Rep. | 22.02 | | [21.38, 22.66] | | 29.72 | [26.46, 33.20] | 31.78 | [30.05, 33.50] |
| Slovakia | 34.61 | [33.73, 35.49] | 42.22 | [40.08, 44.43] | 23.62 | [21.80, 25.44] |  | Slovakia | 38.63 | | [37.08, 39.46] | | 47.28 | [45.15, 49.40] | 27.29 | [25.51, 29.07] |
| Hungary | 26.26 | [25.60, 26.92] | 40.31 | [38.17, 42.45] | 28.91 | [26.90, 30.92] |  | Hungary | 28.12 | | [27.50, 28.74] | | 44.67 | [42.58, 46.76] | 29.86 | [27.97, 31.75] |
| Poland | 23.04 | [22.50, 23.57] | 26.63 | [25.87, 27.39] | 28.15 | [26.18, 30.12] |  | Poland | 23.14 | | [22.63, 23.62] | | 28.06 | [27.34, 28.79] | 30.86 | [28.93, 32.79] |
| Bulgaria | 15.25 | [14.51, 15.99] | 20.74 | [19.07, 22.41] | 13.54 | [12.29, 14.79] |  | Bulgaria | 16.46 | | [15.74, 17.17] | | 25.79 | [24.09, 27.50] | 16.41 | [15.17, 17.66] |
| Romania | 21.05 | [20.35, 21.74] | 22.05 | [21.08, 23.02] | 16.45 | [13.77, 19.13] |  | Romania | 26.25 | | [25.54, 26.97] | | 28.72 | [27.74, 29.70] | 20.5 | [17.75, 23.23] |
| Latvia | 30.06 | [29.09, 31.03] | 47.14 | [45.06, 49.23] | 36.37 | [32.75, 40.00] |  | Latvia | 31.17 | | [30.30, 32.04] | | 50.52 | [48.65, 52.40] | 41.29 | [38.14, 44.43] |
| Lithuania | 24.11 | [23.15, 25.06] | - | - | 31.38 | [28.93, 33.82] |  | Lithuania | 24.81 | | [23.99, 25.64] | | - | - | 38.54 | [36.73, 40.95] |
| Estonia | 32.39 | [31.38, 33.39] | 37.86 | [35.84, 39.88] | 26.33 | [24.39, 28.28] |  | Estonia | 30.76 | | [29.44, 31.16] | | 40.63 | [38.77, 42.48] | 24.22 | [22.51, 25.93] |
| **Total** | 23.36 | [23.22, 23.49] | 26.56 | [26.22, 26.91] | 22.73 | [22.37, 23.10] |  | **Total** | 26.20 | | [26.07, 26.34] | | 30.79 | [30.45, 31.13] | 25.51 | [25.15, 25.87] |
| **^a^** Prevalence rates were standardized using the 2013 European Standard population | | | | | | | | | | | | | | | | |

EHIS – European Health Interview Survey (2006/09); ESS - European Social Survey (2008,2010,2012) ; EU-SILC- EU Statistics on Income and Living Conditions (2008,2012) .

**Online Resource 4**

**Table S4-1. Robustness Analyses using Multi-level Modeling – Comparison of prevalence of GALI disability -Based on country/survey pooled data (ages 30-79) for 28 European countries**

| *Males* | | | | |
| --- | --- | --- | --- | --- |
| ***Survey*** | ***Model A*** | | ***Model B*** | |
|  | ***Main Analysis*** | | ***Multilevel Analysis*** | |
|  | Odds Ratio | 95% CI | Odds Ratio | 95% CI |
| EU-Survey Income Living Conditions (EU-SILC) | 1.00 | ref | 1.00 | ref |
| European Health Interview Survey (EHIS) | **1.27** | [1.15 , 1.41] | **1.49** | [1,46, 1.53] |
|  |  |  |  |  |
|  |  |  |  |  |
| European Health Interview Survey (EHIS) | 0.91 | [0.78, 1.07] | **0.90** | [0.87, 0.92] |
| n | 387,011 | | 387,011 | |
| **Significance In bold (p<0.05)** | | | | |
| *Females* | | | | |
| ***Survey*** | ***Model A*** | | ***Model B*** | |
|  | ***Main Analysis*** | | ***Multilevel Analysis*** | |
| EU-Survey Income Living Conditions (EU-SILC) | 1 | ref | 1 | ref |
| European Health Interview Survey (EHIS) | **1.42** | [1.28 ,1.42] | **1.64** | [1.60, 1.67] |
|  |  |  |  |  |
|  |  |  |  |  |
| European Social Survey (ESS) | 0.92 | [0.78, 1.07] | **0.91** | [0.89, 0.93] |
| n | 445,438 | | 445,438 | |

**Significance In bold (p<0.05)**

Model A : $logit({GALI}_{ikesc})= \beta_{k}Age+ \beta_{s}{Survey}_{s}+ \beta_{e}{Education}_{e}+\beta_{c}{Country}_{c}$ .

Model B : $mqrlogit\left( {GALI}_{ikesc} \right)= \beta_{k}Age+ \beta_{s}{Survey}_{s}+ \beta_{e}{Education}_{e}$ || $\beta_{c}{Country}_{c}$ is estimated as a random intercept

EHIS – European Health Interview Survey (2006/09); ESS - European Social Survey (2008,2010,2012) ; EU-SILC- EU Statistics on Income and Living Conditions (2008,2012) for 28 European countries (Finland, Sweden, Norway, Denmark, UK, Ireland, Netherlands, Belgium, Germany, Austria, Switzerland, France, Spain, Portugal, Italy, Greece, Cyprus, Slovenia, Croatia Czech Republic, Slovakia, Hungary, Poland, Bulgaria, Romania, Latvia, Lithuania, Estonia)

**Table S4-2. Robustness Analyses using Multi-level Modeling – Comparison of educational inequalities Based on country/survey pooled data (ages 30-79) for 28 European countries**

| *Males* | |  |  | |  |  | |
| --- | --- | --- | --- | --- | --- | --- | --- |
| ***Survey*** | | ***Model A*** | | | ***Model B*** | | |
|  |  | ***Main Analysis**** | | | ***Multilevel Analysis*** | | |
|  | | *Odds Ratio* | | *95% CI* | *Odds Ratio* | | *95% CI* |
| EU-Survey Income Living Conditions (EU-SILC) | | 1.00 | | ref | 1.00 | | ref |
| European Health Interview Survey (EHIS) | | **1.41** | | [1.22, 1.64] | **1.67** | | [1.58, 1.76] |
|  |  |  |  |  |  |  |  |
|  |  |  |  |  |  |  |  |
| European Social Survey (ESS) | | 1.13 | | [0.97, 1.31] | **1.14** | | [0.87, 0.92] |
| ***Education*** | | | | | | | |
| High | 1.00 | | | ref | 1.00 | | ref |
| Medium | **1.62** | | | [1.51, 1.74] | **1.69** | | [160-1.69] |
| Low | **2.57** | | | [2.32, 2.85] | **2.62** | | [2.55, 2.70] |
| ***Survey*Education*** | | | | | | | |
| Low*EHIS | **0.81** | | | [0.79, 0.89] | **0.83** | | [0.78, 0.89] |
| Low*ESS | **0.74** | | | [0.58, 0.81]] | **0.68** | | [0.64, 0.73] |
| Medium*EHIS | 0.93 | | | [0.85, 1.03] | **0.91** | | [0.84, 0.95] |
| Medium*ESS | **0.81** | | | [0.68, 0.93] | **0.84** | | [0.78, 0.90] |
| n | 387,011 | | | | 387,011 | | |

EHIS – European Health Interview Survey (2006/09); ESS - European Social Survey (2008,2010,2012) ; EU-SILC- EU Statistics on Income and Living Conditions (2008,2012) ) for 28 European countries.

| *Females* |  |  | |  |  | | |
| --- | --- | --- | --- | --- | --- | --- | --- |
| ***Survey*** | ***Model A*** | | | ***Model B*** | | |  |
|  | ***Main Analysis*** | | | ***Multilevel Analysis*** | | |  |
|  | *Odds Ratio* | | *95% CI* | *Odds Ratio* | | *95% CI* |  |
| EU-Survey Income Living Conditions (EU-SILC) | 1 | | ref | 1 | | ref |  |
| European Health Interview Survey (EHIS) | **1.46** | | [1.19, 1.79] | **1.67** | | [1.59, 1.76] |  |
|  |  |  |  |  |  |  |  |
|  |  |  |  |  |  |  |  |
| European Social Survey (ESS) | 1.04 | | [0.83, 1.30] | **1.15** | | [1.09, 1.21] |  |
| ***Education*** | | | | | | |  |
| High | 1 | | ref |  | |  |  |
| Medium | **1.44** | | [1.36,1.53] | **1.44** | | [1.41, 1.48] |  |
| Low | **2.33** | | [2.07, 2.61] | **2.34** | | [2.29, 2.40] |  |
| ***Survey*Education*** | | | | | | |  |
| Low*EHIS | 0.93 | | [0.74, 1.17] | 0.97 | | [0.92, 1.03] |  |
| Low*ESS | **0.83** | | [0.70, 0.98] | **0.71** | | [0.66, 0.74] |  |
| Medium*EHIS | 0.99 | | [0.86, 1.15] | 0.97 | | [0.91. 1.02] |  |
| Medium*ESS | 0.91 | | [0.82, 1.00] | **0.83** | | [0.78, 0.90] |  |
| n | 445,438 | | | 445,438 | | |  |

**Significance In bold (p<0.05)**

Model A : $logit\left( {GALI}_{ikesc} \right)= \beta_{k}Age+ \beta_{s}{Survey}_{s}+ \beta_{e}{Education}_{e}+\beta_{c}{Country}_{c}+ \beta_{se}{Survey}_{s}*{Education}_{e}$

Model B : $mqrlogit\left( {GALI}_{ikes} \right)= \beta_{k}Age+ \beta_{s}{Survey}_{s}+ \beta_{e}{Education}_{e} + \beta_{se}{Survey}_{s}*{Education}_{e}$ || ${Country}_{c}$ is estimated as a random intercept

EHIS – European Health Interview Survey (2006/09); ESS - European Social Survey (2008,2010,2012) ; EU-SILC- EU Statistics on Income and Living Conditions (2008,2012) ) for 28 European countries. for 28 European countries (Finland, Sweden, Norway, Denmark, UK, Ireland, Netherlands, Belgium, Germany, Austria, Switzerland, France, Spain, Portugal, Italy, Greece, Cyprus, Slovenia, Croatia Czech Republic, Slovakia, Hungary, Poland, Bulgaria, Romania, Latvia, Lithuania, Estonia)

**
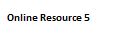
**


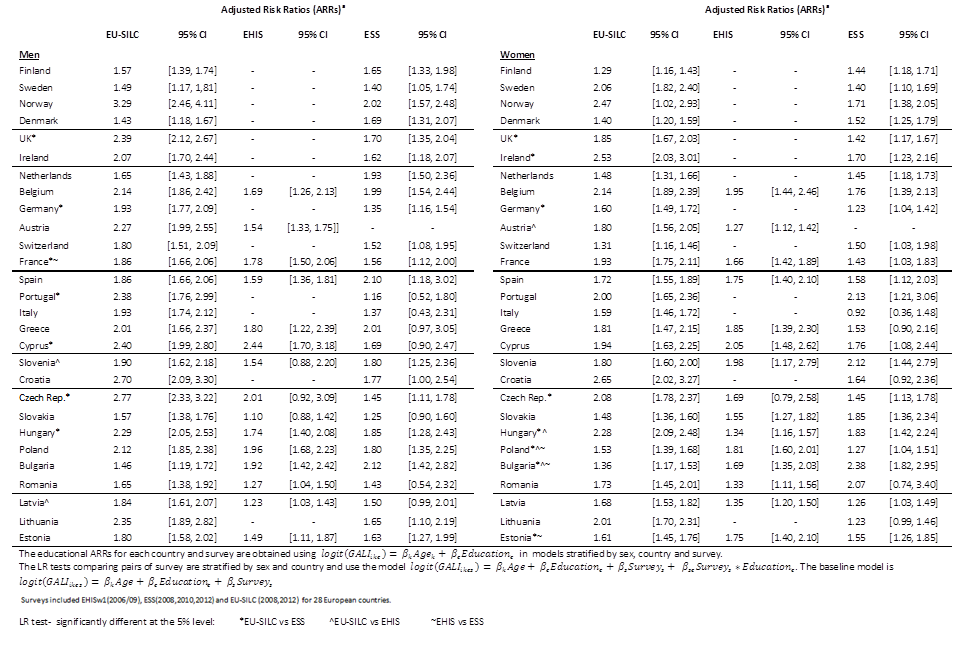


EHIS – European Health Interview Survey (2006/09); ESS - European Social Survey (2008,2010,2012) ; EU-SILC- EU Statistics on Income and Living Conditions (2008,2012) .

**Table S5- Educational Inequalities( Adjusted Risk Ratio) by gender and survey (European Health Interview Survey 2006-09; European Social Survey 2008, 2010, 2012; European Union Statistics on Income and Living conditions 2008, 2012) - Based on country/survey pooled data (ages 30-79) for 28 European Countries**

**Online Resource 6**

**Table S6-1. GALI Phrasing Analysis Restricted to EU-Statistics Living Conditions (2008,2012) - Based on country pooled data (ages 30-79) for 28 European countries**

| ***GALI Phrasing*** | ***Males*** | | ***Females*** | |
| --- | --- | --- | --- | --- |
|  | *Odds Ratio* | *95% CI* | *Odds Ratio* | *95% CI* |
| Standard phrasing | 1.00 | ref | 1.00 | ref |
| No time reference | 1.25 | [0.64, 2.43] | 1.61 | [0.93, 2.77] |
|  |  |  |  |  |
|  |  |  |  |  |
| Personal reference | 1.54 | [1.00, 2.12] | 1.62 | [0.94, 2.09 ] |
| Unknown comparability | 1.14 | [0.80-1,63] | 1.22 | [0.91-1.62] |
| n | 254,986 | | 290,398 | |
| **Significance In bold (p<0.05)** | | | | |

Model: $mqrlogit\left( {GALI}_{ikesc} \right)= \beta_{k}Age+ \beta_{s}{Survey}_{s}+ \beta_{e}{Education}_{e}$ || $\beta_{c}{Country}_{c}$ is estimated as a random intercept. We choose a multi-level model as the GALI phrasing is also collinear with country fixed effects.

Surveys included EHISw1(2006/09), ESS(2008,2010,2012) and EU-SILC (2008,2012) ) for 28 European countries (Finland, Sweden, Norway, Denmark, UK, Ireland, Netherlands, Belgium, Germany, Austria, Switzerland, France, Spain, Portugal, Italy, Greece, Cyprus, Slovenia, Croatia Czech Republic, Slovakia, Hungary, Poland, Bulgaria, Romania, Latvia, Lithuania, Estonia)
